# Effects of long-term low dose saxitoxin exposure on nerve damage in mice

**DOI:** 10.18632/aging.203199

**Published:** 2021-07-01

**Authors:** Qian Sun, Xiao Chen, Wei Liu, Shenpan Li, Yan Zhou, Xingfen Yang, Jianjun Liu

**Affiliations:** 1 Key Laboratory of Modern Toxicology of Shenzhen, Shenzhen Medical Key Discipline of Health Toxicology (2020-2024), Shenzhen Center for Disease Control and Prevention, Shenzhen 518055, Guangdong, People’s Republic of China; 2 School of Public Health, Southern Medical University, Guangzhou 510515, Guangdong, People’s Republic of China

**Keywords:** saxitoxin, neurotoxicity, proteomics, memory, cognitive deficiency

## Abstract

Saxitoxin (STX), as a type of paralytic shellfish poisoning (PSP), is gaining widespread attention due to its long existence in edible shellfish. However, the mechanism underlying STX chronic exposure-induced effect is not well understood. Here, we evaluated the neurotoxicity effects of long-term low-dose STX exposure on C57/BL mice by behavioral tests, pathology analysis, and hippocampal proteomics analysis. Several behavioral tests showed that mice were in a cognitive deficiency after treated with 0, 0.5, 1.5, or 4.5 μg STX equivalents/kg body weight in the drinking water for 3 months. Compared with control mice, STX-exposed mice exhibited brain neuronal damage characterized by decreasing neuronal cells and thinner pyramidal cell layers in the hippocampal CA1 region. A total of 29 proteins were significantly altered in different STX dose groups. Bioinformatics analysis showed that protein phosphatase 1 (Ppp1c) and arylsulfatase A (Arsa) were involved in the hippo signaling pathway and sphingolipid metabolism pathway. The decreased expression of Arsa indicates that long-term low doses of STX exposure can cause neuronal inhibition, which is a process related to spatial memory impairment. Taken together, our study provides a new understanding of the molecular mechanisms of STX neurotoxicity.

## INTRODUCTION

Many toxins can accumulate in edible seafood and cause disease in humans. Marine dinoflagellates and freshwater cyanobacteria can produce a group of paralytic neurotoxins called paralytic shellfish toxins (PSTs). To date, more than 57 PST isomers have been identified, each with different sodium channel affinities and toxic capabilities [1, 2]. Saxitoxin (STX) is a common toxic component of the best-known PST, with the molecular formula C_10_H_17_N_7_O_4_ [3]. The blocking of voltage-gated sodium channels (VGSCs) is responsible for the rising phase of action potentials in neurons, myocytes and other excitable cells [4]. Therefore, STX is a neurotoxin but is also involved in a variety of tissue damage, including liver, kidney, gills, intestines, and muscle tissues [5, 6]. The most severe symptoms of STX poisoning are respiratory paralysis and death.

The environment and its compartments have been frequently polluted by marine toxins. A survey conducted in the North Yellow Sea of China from 2011 to 2012 found the concentration of STX in the range of 30 to 788μg/kg [7]. In the summers of 2018 and 2019, high concentrations of PST were detected in the Southern Bight of the UK, with mean toxicity of 449 μg STX eq./kg [8]. Recently, the concentrations of STX in freshwater reservoirs in Brazil have reached as high as 0.05μM [9]. To protect and improve human health, a standard for STX concentration in drinking water has been established, with the ceiling acceptable level of 0.01μM. However, the underlying mechanism of long-term low-dose exposure of STX-induced nerve damage remains unclear.

Marine dinoflagellates and freshwater cyanobacteria produce a range of toxins including STX [10]. STX has been found in at least 12 species of marine puffer fish and shellfish [11]. Symptoms of STX poisoning are dose-dependent and can appear within ten minutes after oral administration when eating toxin-containing shellfish. The lethal dose (LD50) of STX in mice was 3.4 μg/kg intravenously, 10 μg/kg intraperitoneally and 263 μg/kg orally, respectively. The no observed adverse effect levels (NOAELs) and lowest observed adverse effect levels (LOAELs) are intended to determine the level of exposure at which adverse health effects begin to occur. STX has a LOAEL of 1.5μg STX/kg body weight and a NOAEL of 0.5μg STX/kg body weight based on exposure assessments in mice. The European Commission on Marine Biotoxins (ECBS) scientific opinion on the food chain proposes an acute reference dose (ARfD) of 0.5 μg/kg for STX and its analogs.

STX affects cell components, including DNA, proteins and oxidizes unsaturated lipids, through activating cells to form reactive oxygen species (ROS) [12–14]. Although the acute toxicity of PST has been well studied, the chronic low-dose toxic effects of PST in humans and other animals remain a blank.

The purpose of this study was to address whether chronic asymptomatic STX exposure leads to potential neurotoxicity and health effects in the mammalian system. Therefore, 24-week-old C57BL/6NJ mice were exposed to low asymptomatic doses of STX via drinking water for 3 months to observe behavioral changes and pathological changes in hippocampal regions. The results of this study are applicable to human health.

## MATERIALS AND METHODS

### Animals

Six-month-old C57BL/6NJ mice with an average weight of 25 g were obtained from Guangdong Medical Laboratory Animal Center (China, Guangdong) and domesticated for four weeks. All mice were housed in a 12-hour light and dark cycle at 22 ± 2° C at the Shenzhen Center for Disease Control and Prevention and Prevention (SZCDC) and received a standard rodent diet. Experiments were performed according to the principles of the NIH nursing guidelines. The experimental animals were approved by the Ethics Committee of the SZCDC.

Mice were divided into four groups (n=16), and the control group was given a standard diet and drinking water. Three dose groups were used to evaluate the neurotoxicity of STX: the lowest dose group (NOAEL, 0.5 μg STX/kg body weight), the equivalent triple dose of the medium-dose group (LOAEL, 1.5 μg STX/kg body weight), and the high dose group (4.5 μg STX/kg body weight), which are marked as CT, Low, Middle, and High, respectively. STX was purchased from the National Research Council (NRC) of the National Measurement Standards Institute of Canada. STX is easily soluble in water. Freshly prepared aqueous STX solution in proportion every other day. Mice were exposed to STX via drinking water for three months. Subsequently, several behavioral tests were performed on nine-month-old mice to assess their cognitive function. At the end of the behavioral experiments, the mice were sacrificed. The hippocampus was dissected and appropriately processed for further analysis.

### Behavioral tests

Considering the interaction between the experiments, the mice were sequentially subjected to step-down passive avoidance test (SDPA) and Morris water maze test (MWM), respectively. There was a 2-day interval between designed behavioral tests. Behavioral tests were performed between 9:00 and 17:00 in an air-regulated and soundproof experimental laboratory. After testing, the apparatus was cleaned with 70% ethanol and water to remove olfactory traces.

### SDPA test

The SDPA test was performed to assess state-dependent learning and short memory of mice [15]. The device consists of a wire cage (length 75 cm × width 75 cm × height 46 cm) and a layer of stainless steel rods, which are divided into five equal chambers with 0.8cm intervals. Then, in the middle of the floor, a 4.5 cm plastic platform was positioned. Mice (n = 12 per group) were sequentially subjected to two experimental trials: a learning phase and a 24-hour washing period. During the learning period, mice were placed on a floor and given a 5-minute electric shock (36V, AC). The mouse was considered to remember the shock if it was left on the plastic platform for at least 3 minutes. Mice that stayed on the platform at least twice for three minutes were excluded. During the short memory retention session, the buck delay and the number of errors were recorded for 5 minutes.

### MWM test

MWM test was performed to evaluate spatial memory and learning according to the previous method [15]. The water maze apparatus consisted of a circular pool (170 cm in diameter and 30 cm in height) filled with an opaque liquid (a white color made from milk). A circular white escape platform (diameter: 10 cm) was placed 1 cm below the surface of the water. Navigation tests and probe tests were performed on 12 mice in each group.

For the next 5 days (navigation trials), the mice were trained to find the hidden platform for four times 90-second trials per day. After gently guiding them to the platform, they stayed there for 15 seconds and then returned to the cage. If they cannot find the platform themselves, they would be manually placed on the platform. Probe test trials were conducted 6 days after the completion of the navigation trials.

In the probe test, mice were allowed to swim in a swimming pool without an escape platform for 2 minutes. Various parameters were recorded, including escape latency, the number of crossings, time spent in the target quadrant, and distance traveled in the target quadrant.

### Proteomic analysis

### Protein samples preparation

The mice hippocampi protein samples were prepared based on the previous method [16]. Briefly, mice were anesthetized by intraperitoneal injection of 60 mg/kg sodium pentobarbital and then executed. Various tissues, including hippocampal tissue, were dissected. Some of these tissues were stored at 80° C until it was used, while others were placed in paraformaldehyde. Proteins were extensively extracted from hippocampal tissues with an 8 M urea-PBS solution. The tissues were then lysed and sonicated using an ultrasonic homogenizer (Sonics and MaterialsInc., Newtown, CT, USA). The cell lysates were centrifuged and the protein concentration in the supernatant was determined using a NanoDrop 2000/2000c spectrophotometer (Thermo Fisher Scientific, Waltham, MA, USA).

### TMT labeling

100 μg samples were denatured with dithiothreitol (DTT) and then treated with iodoacetamide (IAA). Protein samples were diluted with PBS and then digested with Lys-C (1:100, w/w) for 2 h at 25° C, followed by further digested with trypsin (1:50, w/w) overnight at 37° C and acidified with 1% formic acid (Thermo Fisher Scientific, Waltham, MA, USA). The supernatants were desalted by centrifugation with a reversed-phase column (Oasis HLB; Waters, Milford, MA, USA), dried with a vacuum concentrator, and then dissolved in triethylammonium bicarbonate (Applied Biosystems, Milan, Italy). Peptides and TMT reagents were vortexed and incubated for 1 h at room temperature: hippocampi of the CT (TMT-126), hippocampi of the Low (TMT-127), hippocampi of the Middle (TMT-128), hippocampi of the High (MT-129) (n = 6 per group). Hydroxylamine was used to terminate the reactions. Before high performance liquid chromatography (HPLC) analysis, equal amounts of labeled peptides of different moieties were mixed and desalted in 100 μL 0.1% formic acid.

### HPLC

TMT-labeled peptides were fractionated using the previously described method [17]. Peptides were loaded directly onto the Xbridge BEH300 columns for the C18 analytical runs (Waters, Milford, MA, USA) followed by HPLC separation (UltiMate 3000 UHPLC; Thermo Fisher Scientific, Waltham, MA, USA). Scores were collected serially. Then, dissolved in 20 μL of 0.1% formic acid for LC-MS/MS analysis.

### LC-MS/MS analysis and database searching

Peptide analysis was conducted by LC-MS/MS method, following previous methods [16]. Soon, the final 3000RSLCnano system was precisely interfaced with a ThermoQ Exactive Benchtop mass spectrometer (both from Thermo Fisher Scientific, Waltham, MA, USA). Peptides were first loaded onto a capture column and then separated with an analytical capillary column (Upchurch, Oak Harbor, WA, USA) packed with C18 silicone (Varian, Lexington, MA, USA). Raw mass spectra were searched on the Uniprot Muscle Sequence Database (released in October 2018) using Proteome Discoverer 2.1 software (Thermo Fisher Science, Waltham, MA, USA).

### Bioinformatics analysis and prediction

Differentially expressed proteins were determined (requirements: adjusted *P* < 0.05, fold change < 0.83 or > 1.2). Bioinformatic analysis was performed using the OmicShare tools at https://www.omicshare.com/tools. All proteins identified in the mouse hippocampus were characterized using DAVID Bioinformatics Resources 6.8 (https://david.ncifcrf.gov/) and Omic Share tool (https://www.omicshare.com/tools) for molecular function (M F), cellular component (CC), biological process (BP), and Kyoto Encyclopedia of Genes and Genomes (KEGG) pathway classification. Protein-protein interactions were analyzed using the STRING database (https://string-db.org/).

### Western blot (WB)

Hippocampal proteins were extracted from each group using lysis buffer (Beyotime, China) and phosphatase inhibitor cocktail (Thermo Fisher Science, USA). Protein samples were mixed with loading buffer, heated at 100° C for 5 min, then separated on 10% SDS-PAGE and transferred to PVDF membranes. Then, blots were blocked with 5% nonfat dried milk for 2 h at room temperature. Monoclonal anti-Ppp1c (diluted 1 : 2000, molecular weight: 39 kDa, Abcam, USA), monoclonal anti-Arsa (diluted 1 : 2000, molecular weight: 54 kDa, Abcam, USA), anti-Tau (phospho S262 + T263) (diluted 1 : 2000, molecular weight: 79 kDa, Abcam, USA), anti- total Tau (diluted 1 : 2000, molecular weight: 52 kDa, Abcam, USA) or monoclonal anti-β-actin (diluted 1 : 1000, molecular weight: 42 kDa, Santa Cruz, USA) were used as primary antibodies. Anti-rabbit or anti-mouse IgG HRPs (diluted 1: 3000, Thermo Fisher Scientific, USA) were used as secondary antibodies. After washing in TBST, the membranes were exposed to chemiluminescence reagents using an electronic chemiluminescence kit on a phosphor imager (Pierce Biotechnology, USA) and analyzed for optical density based on Java (ImageJ) software (National Institutes of Health, Bethesda, MD, USA).

### Hematoxylin and eosin (HE) staining

Histological analysis of the whole mouse brain was performed. Paraffin-embedded brain sections 4μm were dewaxed three times in xylene for 20min, then rehydrated in 100% ethanol for 5min, 95% ethanol for 5 min, 80% ethanol for 5min, and then dehydrated three times in PBS for 5 min. Cell nuclei were counterstained with hematoxylin for 5 min, then rinsed with tap water for 1 min. After treated with 1% hydrochloric acid alcohol solution for 10 s, the sample was rinsed with tap water for 1 min and distilled water for 15 min. Finally, the sections were counterstained with 0.5% eosin dye for 2 min and immediately dehydrated. The dehydrated sections were observed under a light microscope.

### Statistical analysis

Statistical analysis was carried out using GraphPad Prism 8 (GraphPad Software, La Jolla, CA, USA). Derived values are presented as the mean ± the standard error of the mean (SEM). Statistical differences between treatment groups and the CT group were carried out by a one-way analysis of variance (ANOVA) followed by Turkey's post-hoc test, and a multiple-range least significant difference was used for intergroup comparisons. Differences were considered statistically significant at *p* < 0.05.

## RESULTS

### STX impaired cognitive performance of mice

**Figure 1:**
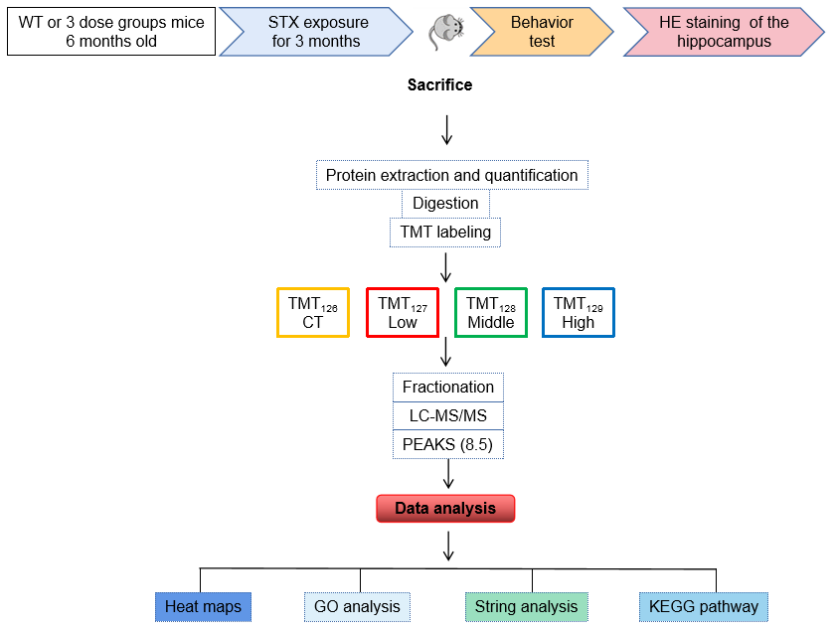
**Flow-chart of experimental design.** C57BL/6NJ mice were treated with 0, 0.5, 1.5 or 4.5 μg Saxitoxin (STX) equivalents/kg body weight in the drinking water for 3 months. The mice were sacrificed after behavioral tests, and brain hippocampal tissues were isolated for further HE staining and proteomic analysis. Protein was pooled from six animals in each group (Marked as CT, Low, Middle, High) and was digested into peptides. The peptides were further labeled with 6-plex TMT and HPRP fractionation followed by LC-MS/MS analysis. Proteomics analysis was performed by using PEAKS 8.5 software, and bioinformatics analysis. Including heat map, GO, STRING and KEGG pathway was were performed using DAVID 6.8 and OmicShare.

In the SDPA test, compared with CT mice, STX-exposed mice exhibited a shorter step-down latency and more errors in a dose-dependent relationship (Figure 1A, 1B). The results of the MWZ test showed that STX-exposed mice took longer to reach the platform (escape latency) during the training period than CT mice (Figure 1C). During the trial period, the platform was removed and the frequency of mice crossing the position of the target platform was counted. The search time of mice in the correct quadrant was recorded to assess whether there was a difference in training time between different doses of STX exposure. The swimming trajectory of mice in the target quadrant was shown in (Figure 1D). As shown in Figure 1E–1H, STX-exposed mice took much longer to find the target quadrant, crossed the platform less frequently, and spent less time in the correct quadrant than that in CT mice. Taken together, our data suggested that STX impaired the cognitive performance of mice.

### Effects of STX exposure on the pyramidal cells and neuronal cells in the CA1 area of the hippocampus

**Figure 2:**
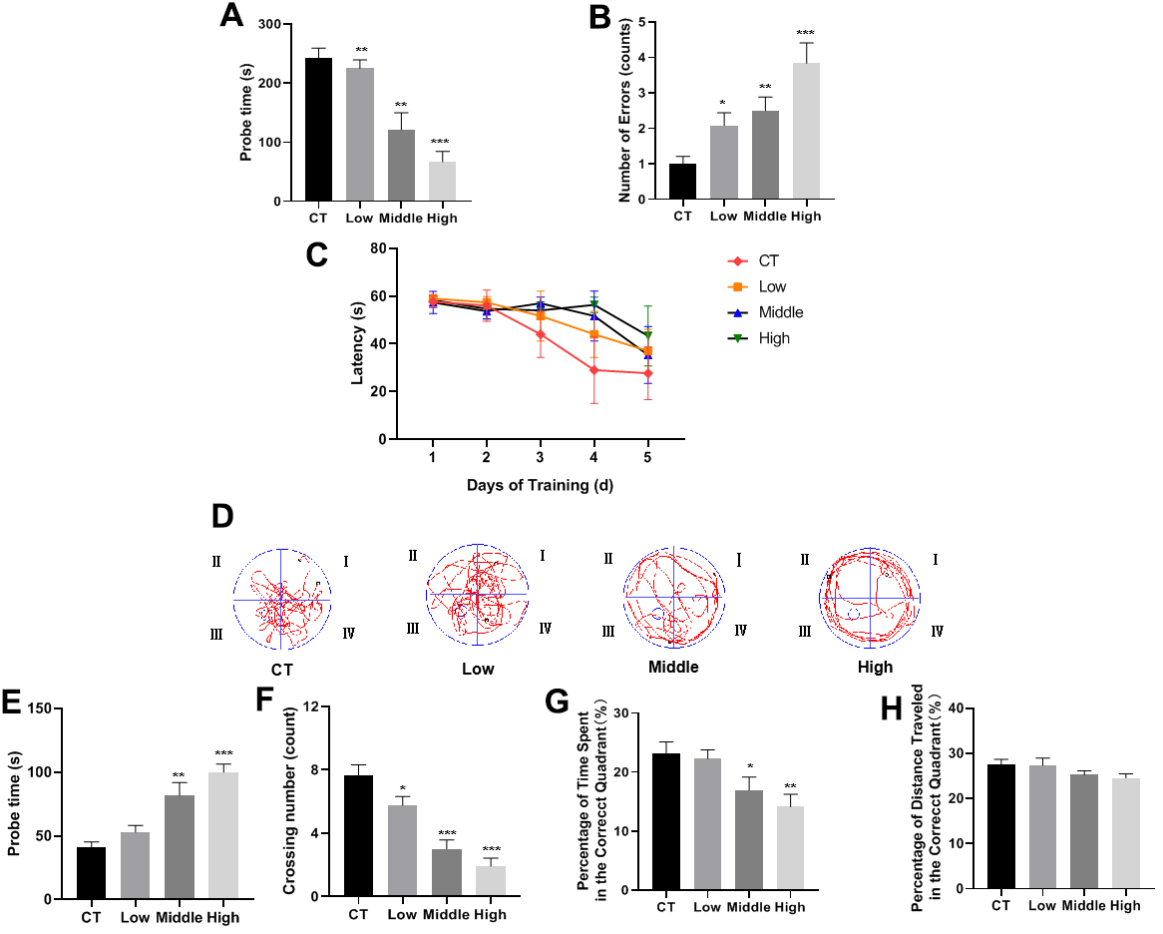
**STX impaired cognitive performance of the mice.** (**A**, **B**) STX significantly reduced the step-down latency and increased the number of errors made by the step-down passive avoidance (SDPA) test. STX aggravated spatial learning and memory deficits in C57BL/6NJ mice assessed by the Morris water maze (MWM) test. (**C**) The escape latency of mice in a training session from day 1 to day 5. (**D**) The swimming trajectory of mice during the probe test. (**E**–**H**) Differences in probe time, the number of crossing movements, percent of time spent in the platform quadrant, and percent of distance travelled in the platform quadrant in the probe trial of the MWM test. Bar graphs show mean {plus minus} SEM, (n=12 for each group), *p<0.05, **p<0.01 or ***p<0.001 vs. CT group by two-tailed unpaired Student's t-test.

The hippocampus is highly vulnerable and sensitive to STX. HE staining revealed that pyramidal cell and neuronal cell layers were densely and regularly arranged in CA1 subregions of the CT group, while granulovacuolar degeneration was observed in the CA1 subregion of the hippocampus of the STX exposed group (Figure 2). With increasing doses of STX exposure, in the CA1 region, the pyramidal cell layer became thinner, cell arrangement was disorganized, the cell morphology was irregular, and the pyknotic cells in the pyramidal cell layers increased. Furthermore, the neuronal cells decreased in CA1 regions of STX-exposed mice, proving that hippocampal neuronal loss was induced by STX.

### Effects of STX exposure on the hippocampal proteome of mice

**Figure 3:**
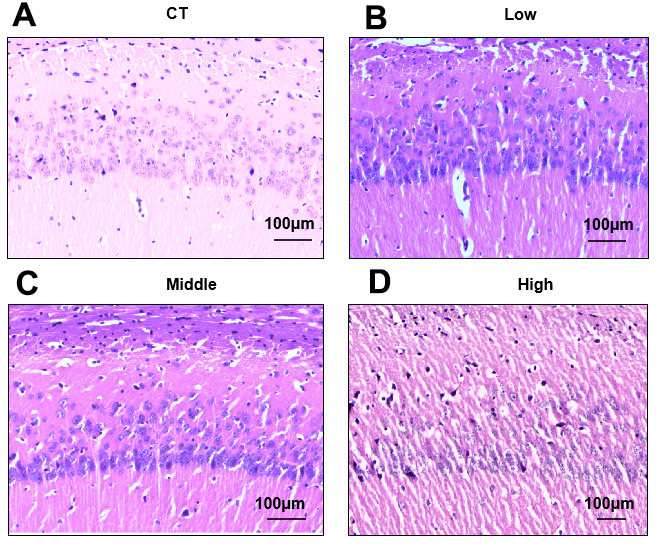
**Effects of STX exposure on the pyramidal cells and neuronal cells in the CA1 area of the hippocampus.** (**A**–**D**) Representative photomicrographs of Hematoxylin-Eosin (HE) staining of brain sections (n = 6 for each group) from CT, Low, Middle and High group. Magnification, 100×. Scale bar, 100 μm.

The flow-chart of experimental design was shown in Graphical Abstract. Hippocampal proteomics was analyzed by using a 6-plex TMT labeling quantitative proteomics strategy. A total of 6310 proteins were identified by one or more unique peptides (false discovery rate (FDR) <1%). Among the altered proteins in the hippocampus, 69 proteins were significantly altered in the hippocampal tissues in the low dose STX exposure (Low) group (adjusted P < 0.05 and ratio < 0.83 or > 1.2) (Figure 3A). Among the 69 differentially expressed proteins, 37 proteins were significantly down-regulated in Low compared with CT, and 32 proteins were up-regulated. In addition, 87 proteins were affected in the hippocampal tissues of middle dose STX exposure (Middle) group (adjusted *p* < 0.05 and ratio < 0.83 or > 1.2) (Figure 3B). Among 87 differentially expressed proteins, down-regulated proteins were 59 in the Middle compared with CT and 28 proteins were up-regulated. In the high dose STX exposure (High) group, 86 proteins were significantly modulated compared with CT (32 up-regulated, 54 down-regulated) (adjusted *p* < 0.05 and ratio < 0.83 or > 1.2) (Figure 3C). The information about the differentially expressed proteins is detailed in Supplementary Tables 1–3.

### Bioinformatics analysis of differential expression proteins

**Figure 4:**
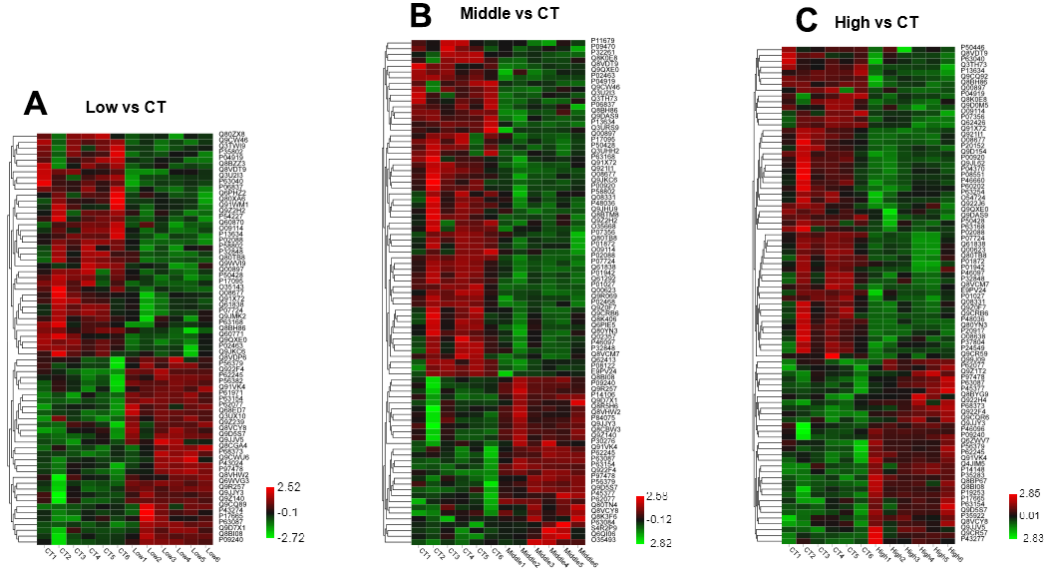
**Heat map of altered proteins induced by STX treatment in mice.** (**A**) Hierarchical clustering of 70 changed proteins in the hippocampus between Low dose STX exposure group and CT group. (**B**) Hierarchical clustering of 87 changed proteins in the hippocampus between Middle dose STX exposure group and CT group. (**C**) Hierarchical clustering of 86 changed proteins in the hippocampus between High dose STX exposure group and CT group. (criteria: ratio {greater than or equal to}1.2 represented up-regulation or ratio <0.83 represented down-regulation). The different color stands for a different level of protein expression, with red indicates increase and green indicates decrease when compared with CT group.

DAVID and OmicShare tools, gene ontology (GO) analysis was applied to classify the differential expression of hippocampal proteins. Proteins significantly altered in the hippocampus were classified according to biological processes (BPS), molecular functions (MFs), and cellular components (CCs). Figure 4 was displayed with the enriched items according to the corresponding *p*-values and -Log2 (Fold Change). The BPs of the identified proteins mainly was involved cellular process, regulation of the biological process, metabolic process and so on. The results for MFs showed the strong enrichments of binding, catalytic activity, transporter activity and molecular function regulation even in different STX exposure groups. CCs displayed the strong enrichments of cell part, organelle, membrane, synapse and so on.

### KEGG pathway analysis of proteins that were changed in different dose STX exposure groups

**Figure 5:**
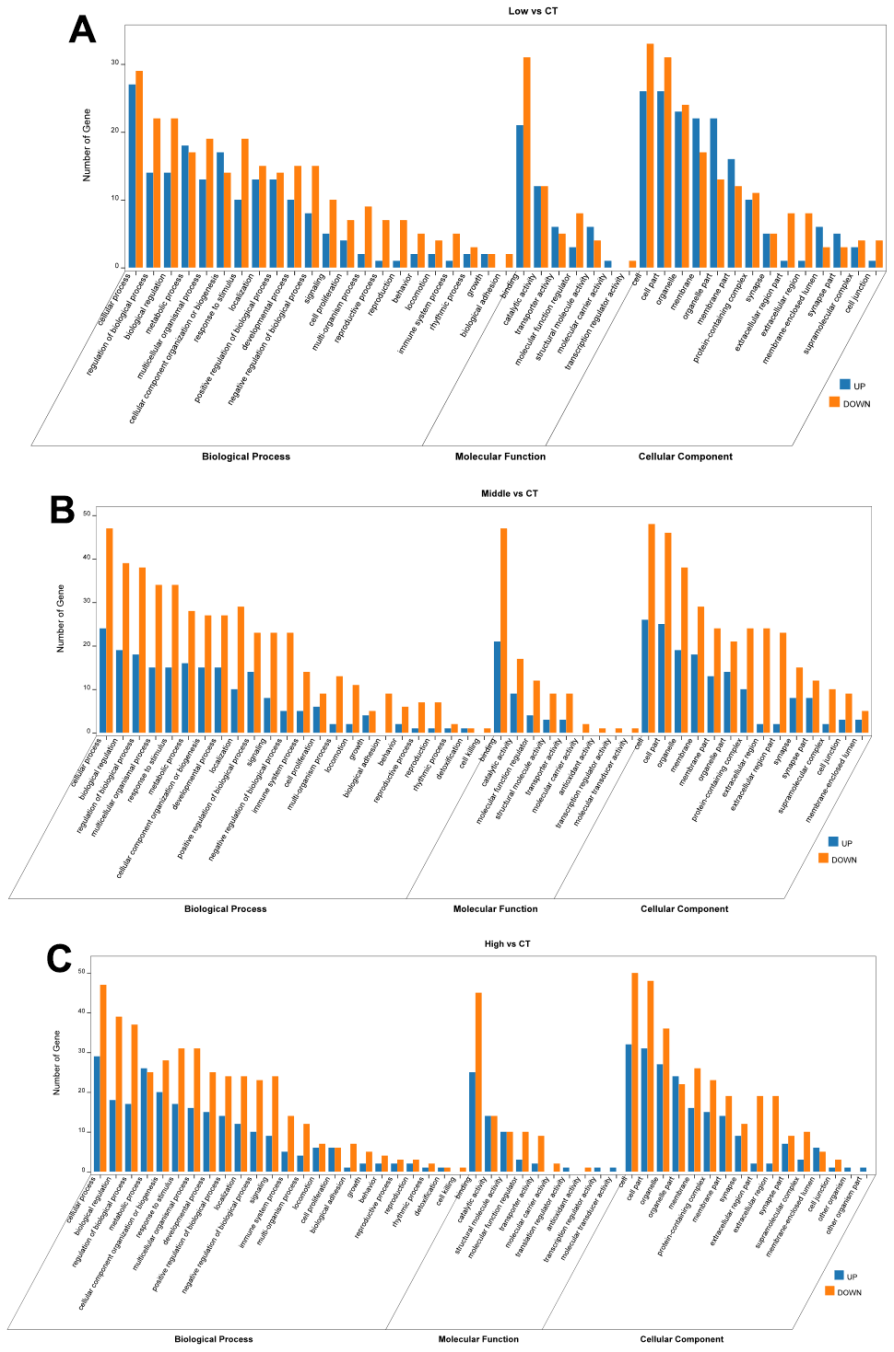
**OmicShare Gene Ontology (GO) enrichment analysis of biological process (BPs), molecular functions (MFs) and cellular component (CCs) for the differentially expressed proteins in the hippocampus.** (**A**) Low dose STX exposure group vs CT group. (**B**) Middle dose STX exposure group vs CT group. (**C**) High dose STX exposure group vs CT group. and listed according to the -Log2 (Fold Change).

To investigate the effect of different doses of STX on hippocampal protein expression, we performed a Venn analysis shown in Figure 5A. A total of 29 proteins were significantly changed in different STX dose groups (Table 1). GO analysis of these 29 proteins was shown in Figure 5B. KEGG pathway analysis revealed that the highly enriched terms included sphingolipid metabolism, D-Glutamine and D-glutamate metabolism, hippo signaling pathway and other top 20 pathways (Figure 5C). Among the 29 proteins, only 14 proteins had interaction with each other (Figure 5D).

**Table 1:** 29 proteins were significantly changed in the different STX dose groups hippocampal formation plays an important role in processes such as memory and learning.

**Accession**	**Description**	**Student's T-test difference low_ct**	**Student's T-test difference middle_ct**	**Student's T-test difference high_ct**
O08677	Kininogen-1 OS=Mus musculus OX=10090 GN=Kng1	-0.352078	-0.485358	-0.371883
O09114	Prostaglandin-H2 D-isomerase OS=Mus musculus OX=10090 GN=Ptgds	-0.439215	-0.340374	-0.379188
P02088	Hemoglobin subunit beta-1 OS=Mus musculus OX=10090 GN=Hbb-b	-0.452371	-0.626415	-0.532594
P04919	Band 3 anion transport protein OS=Mus musculus OX=10090 GN=Slc4a1	-0.465134	-0.482751	-0.415192
P07724	Serum albumin OS=Mus musculus OX=10090 GN=Alb	-0.301107	-0.54441	-0.517958
P09240	Cholecystokinin OS=Mus musculus OX=10090 GN=Cck	0.473583	0.390309	0.376633
P13634	Carbonic anhydrase 1 OS=Mus musculus OX=10090 GN=Ca1	-0.353054	-0.478792	-0.514385
P32848	Parvalbumin alpha OS=Mus musculus OX=10090 GN=Pvalb	-0.436254	-0.379059	-0.391542
P50428	Arylsulfatase A OS=Mus musculus OX=10090 GN=Arsa	-0.318083	-0.286073	-0.285797
P56379	ATP synthase subunit ATP5MPL, mitochondrial OS=Mus musculus OX=10090 GN=Atp5mpl	0.32243	0.321107	0.411315
P62077	Mitochondrial import inner membrane translocase subunit Tim8 B OS=Mus musculus OX=10090 GN=Timm8b	0.515972	0.383606	0.374301
P62245	40S ribosomal protein S15a OS=Mus musculus OX=10090 GN=Rps15a	0.306509	0.307658	0.378685
P63087	Serine/threonine-protein phosphatase PP1-gamma catalytic subunit OS=Mus musculus OX=10090 GN=Ppp1cc	0.420552	0.34901	0.291468
P63154	Crooked neck-like protein 1 OS=Mus musculus OX=10090 GN=Crnkl1	0.556272	0.398606	0.274537
P63168	Dynein light chain 1, cytoplasmic OS=Mus musculus OX=10090 GN=Dynll1	-0.472216	-0.372471	-0.371219
P97478	5-demethoxyubiquinone hydroxylase, mitochondrial OS=Mus musculus OX=10090 GN=Coq7	0.377863	0.286941	0.288508
Q00897	Alpha-1-antitrypsin 1-4 OS=Mus musculus OX=10090 GN=Serpina1d	-0.361168	-0.389279	-0.304951
Q61838	Pregnancy zone protein OS=Mus musculus OX=10090 GN=Pzp	-0.303519	-0.538264	-0.394583
Q80TB8	Synaptic vesicle membrane protein VAT-1 homolog-like OS=Mus musculus OX=10090 GN=Vat1l	-0.304523	-0.511452	-0.336342
Q8BH86	D-glutamate cyclase, mitochondrial OS=Mus musculus OX=10090 GN=Dglucy	-0.280367	-0.266742	-0.269957
Q8BI08	Protein MAL2 OS=Mus musculus OX=10090 GN=Mal2	0.278012	0.268981	0.29278
Q8VCY8	Phospholipid phosphatase-related protein type 2 OS=Mus musculus OX=10090 GN=Plppr2	0.362178	0.300622	0.287837
Q8VDT9	39S ribosomal protein L50, mitochondrial OS=Mus musculus OX=10090 GN=Mrpl50	-0.624302	-0.506995	-0.403882
Q91VK4	Integral membrane protein 2C OS=Mus musculus OX=10090 GN=Itm2c	0.313347	0.277677	0.43629
Q91X72	Hemopexin OS=Mus musculus OX=10090 GN=Hpx	-0.322242	-0.457708	-0.46783
Q922F4	Tubulin beta-6 chain OS=Mus musculus OX=10090 GN=Tubb6	0.359468	0.436015	0.405984
Q9D5S7	Leucine-rich repeat and guanylate kinase domain-containing protein OS=Mus musculus OX=10090 GN=Lrguk	0.451202	0.312099	0.371233
Q9JJY3	Sphingomyelin phosphodiesterase 3 OS=Mus musculus OX=10090 GN=Smpd3	0.323244	0.289372	0.295369
Q9QXE0	2-hydroxyacyl-CoA lyase 1 OS=Mus musculus OX=10090 GN=Hacl1	-0.610957	-0.415358	-0.339566

Keep reading to learn more about its structure and functions.

The sphingolipid metabolism pathway is closely associated with the development of AD [18, 19]. The results show the different expression proteins involved in the Sphingolipid metabolism pathway. The expression of arylsulfatase A (Arsa) was down-regulated and sphingomyelin phosphodiesterase 3 neutral (Smpd3) was up-regulated in the STX-treated mice compared with the CT. With this, other pathways are mostly related to metabolic pathways, suggesting that the effects of STX on the nervous system may be achieved through metabolism.

### Confirmation of differentially expressed protein

Proteomic analysis suggests that the aberrant expression of metabolism-related proteins may be involved in STX induced nervous system damage. Further validation of differentially expressed proteins in proteomics data was performed using Western blot. Based on bioinformatics analysis Arsa (Uniport accession, P50428) and Ppp1cc (Uniport accession, P63087) were selected to be further analyzed.

**Figure 6:**
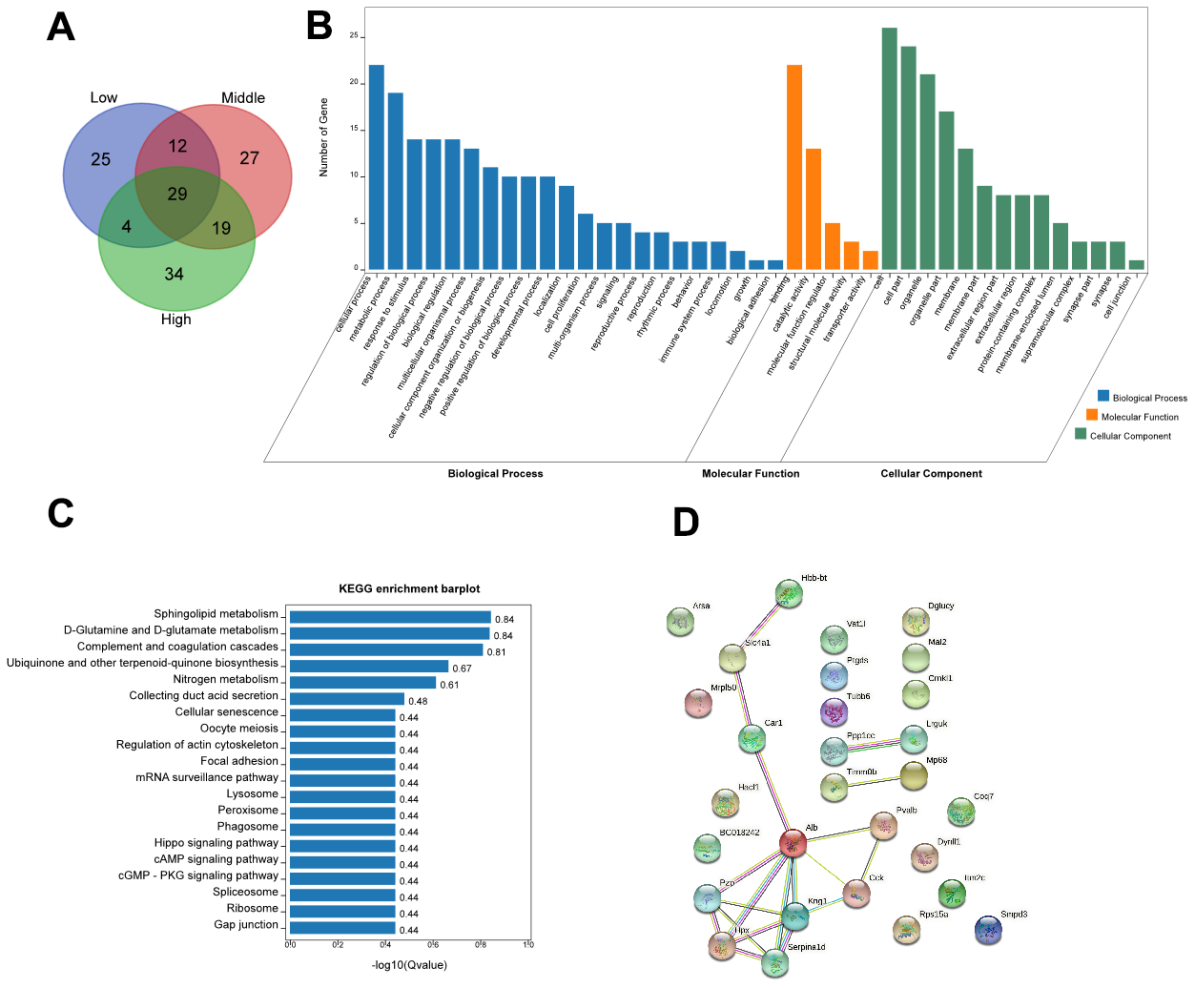
**Bioinformatic analysis.** (**A**) The Venn diagram showed a total of 29 proteins that were significantly changed in the different STX dose groups. (**B**) OmicShare GO enrichment of 29 commonly differentiated proteins. (**C**) Kyoto Encyclopedia of Genes and Genomes (KEGG) analysis of 29 commonly differentiated proteins. (**D**) STRING diagram showing the protein-protein interaction network of the 29 commonly differentiated Proteins.

Metabolic changes in sphingolipid have been related to various neurodegenerative diseases, including AD and Parkinson's disease (PD), which are caused by deficient activity of Arsa [20, 21]. Protein phosphatase 1, catalytic subunit, gamma isoform (Ppp1cc) protein are associated with multiple signaling pathways, including cAMP signaling pathway, Hippo signaling pathway, long-term potentiation, Insulin signaling pathway, etc. Some studies have pointed out that inhibition of protein phosphatase 1 can stimulate the secretion of amyloid precursor protein [22]. The results of the proteomic analysis were verified by Western blot, showing that expression of Arsa protein was down-regulated and Ppp1cc protein was up-regulated in the STX-exposed mice in a dose-dependent relationship compared to the CT (Figure 6).

**Figure 7:**
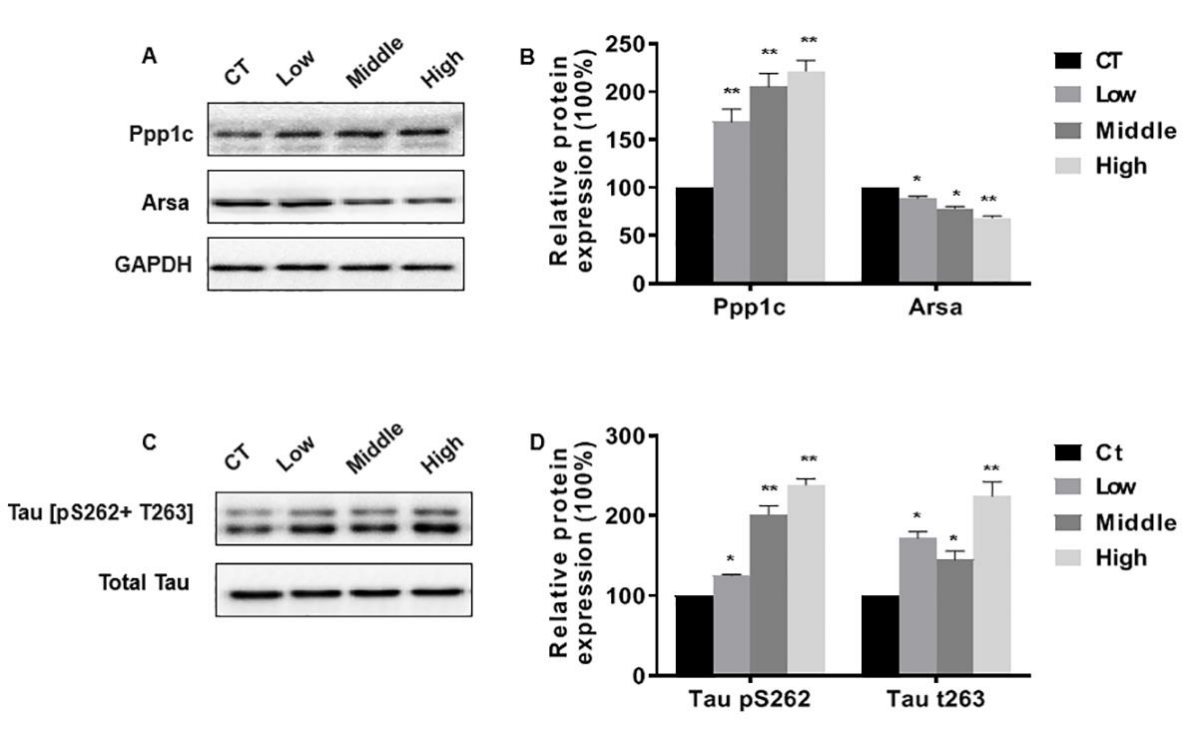
**Confirmation of differentially expressed protein.** (**A**–**C**) Western blots were performed, and (**B**–**D**) relative protein levels of Ppp1c, Arsa and tau[pS262+ T263] were measured. Blots were quantified by densitometry and normalized by use of GAPDH or Total Tau to correct for differences in loading of proteins. The graphed results depict the mean {plus minus} SEM. *P < 0.05, **P < 0.01 versus the control mice (n = 6 for each group).

Tau protein hyperphosphorylation is associated with abnormal expression of Ppp1cc. STX exposure had no effect on the expression of total Tau protein in the hippocampi of C57BL/6NJ mice, the levels of hippocampal tau[pS262+ T263] were significantly increased after long-term low- dose STX exposure exposure(Figure 7).

## DISCUSSION

PSTs are often referred to as paralytic shellfish poisons (PSP), of which STX is one of the most prominent. The prevalence of STX in natural marine products is likely to increase as harmful algal blooms [23, 24]. Current China and International seafood safety regulations only target high-level STX exposure and protect shellfish consumers from acute high dose STX exposure characterized by tingling, numbness, burning of the perioral region, ataxia, giddiness, drowsiness, fever, rash, staggering and even death [25]. In conclusion, the mice model was used to explore chronic low-level STX exposure risks and underlying neurotoxic mechanisms in this study.

Our findings on cognitive behavior and hippocampal proteomics in mice exposed to asymptomatic STX suggest that there is an urgent need to consider the risk of chronic low-level STX exposure.

Previous studies have emphasized the influence of STX in hepatotoxicity, neurotoxicity, and gastrointestinal irritation [13, 26, 27]. As research progressed, it was noted that STX was associated with behavioral alteration and spatial memory loss [28]. The purpose of this study was to investigate whether low-level exposure to STX for 3 months could significantly affect cognition and alter the expression of hippocampal tissue proteins in a mammalian model system.

The intraperitoneal LD50 of STX is 5-10 μg kg^−1^ in mice [29]. Acute STX exposure in rodents can cause neurobehavioral changes such as scratching, gagging, loss of balance control, tremors, and gross histologic lesions in the brain. Previous studies in Wistar rats have investigated the effects of 9μg/L exposures to STX via drinking water for 30 days and find an amnesic effect of STX on aversive and spatial memories [28]. However, chronic low-dose STX exposure studies are still very inadequate. To our knowledge, our study is the first study to investigate the effects of long-term low-level STX exposure on neurological damage in a mammalian model. Spatial memory and learning ability in mice were observed through several behavioral tests, demonstrating that long-term low-level STX exposure could induce cognitive impairments of the mice (Figure 2). Additionally, HE staining validated that long-term low-level exposures did not cause significant gross morphological lesions in hippocampal regions of the brain, but decreased pyramidal cells and neuronal cells in CA1 (Figure 3). These results were consistent with behavioral studies in which long-term low-level STX exposure induced cognitive impacts.

The evidence for STX in the context of neurodegeneration is limited. Melegari et al [30] have reported that STX is able to induce oxidative stress in algae and neural cell cultures through the lipid peroxidation (LPO) pathways. As we know, LPO mechanisms are well established in the pathogenesis of AD [31]. To further explore the mechanism of STX causing neurodegenerative diseases, proteomics has been applied. In our study, we found that the expression of 29 proteins was changed in different dose groups. Bioinformatics analysis of those proteins showed that STX promoted the expression levels of sphingolipid metabolism related protein Smpd3, and inhibited the expression of Arsa. The sphingolipid metabolism pathway is closely associated with the development of AD [18, 19]. Furthermore, STX altered the expression of Alzheimer's disease-related protein complexin 1(Cplx1), especially at a dose of 0.5 μg STX equivalents/kg body weight. Abnormal expression of cplx1 is also associated with a variety of neurological diseases, such as Behçet's disease [32] and PD [33]. Interestingly, several mitochondrial proteins involved in AD, PD and Huntington's disease were only significantly changed in the chronic low dose group, such as ATP synthase subunit epsilon (P56382), Cytochrome c oxidase subunit 7C (P17665), Cytochrome c oxidase subunit 6A1 (P43024). The proteomics results indicated that STX induced alterations in hippocampal proteins, which exhibited dysregulation of proteins involved in the metabolism process, response to stimulation, and behavior. However, the timing and method of STX exposure in this study differed from human exposure to STX in the environment. Therefore, additional studies are needed to assess the potential risks of chronic low-level exposure to STX and to verify the molecular mechanism of STX-induced never damage.

## CONCLUSIONS

In this study, a mouse model was used to explore the underlying toxic mechanism of long-term low-level STX exposure. The present study demonstrated that daily exposure for 3 months to low levels of STX could cause significant cognitive deficits and neuronal cell cutbacks. The alterations of hippocampal sphingolipid metabolism and hippo signaling pathway related proteins may be involved in the STX-induced never damage. These findings provide new insights and evidence for STX neurotoxicity. But, further studies are needed to elucidate the role of the identified key proteins in STX-mediated cognitive impairment and the associated molecular mechanisms.

### Ethical approval

All applicable international, national, and/or institutional guidelines for the care and use of animals were followed.

## Supplementary Material

Supplementary Table 1

Supplementary Table 2

Supplementary Table 3
