# Supplementary material for: Effects of long-term low dose saxitoxin exposure on nerve damage in mice
Source: Aging (Albany NY). 2021 Jul 1;13(13):17211–26. doi: 10.18632/aging.203199 (PMC8312470; doi:10.18632/aging.203199)
Supplement: Supplementary Table 1 [file aging-13-203199-s001.docx]

**Supplementary Table 1. 69 proteins differentially expressed in low compared with CT.**

| **Accession** | **Description** | **Sum PEP Score** | **Coverage** | **Fold Change low_ct** | **p-value low_ct** | **q-value low_ct** |
| --- | --- | --- | --- | --- | --- | --- |
| Q60771 | Claudin-11 OS=Mus musculus OX=10090 GN=Cldn11 PE=1 SV=1 | 38.1287392 | 10.14492754 | 0.644353817 | 0.00375168 | 0.0986222 |
| Q8VDT9 | 39S ribosomal protein L50, mitochondrial OS=Mus musculus OX=10090 GN=Mrpl50 PE=1 SV=2 | 6.46005618 | 15.09433962 | 0.64873357 | 0.00258711 | 0.0824348 |
| Q9QXE0 | 2-hydroxyacyl-CoA lyase 1 OS=Mus musculus OX=10090 GN=Hacl1 PE=1 SV=2 | 9.034036012 | 4.647160069 | 0.654762227 | 6.38E-05 | 0.0160921 |
| Q9CW46 | Ribonucleoprotein PTB-binding 1 OS=Mus musculus OX=10090 GN=Raver1 PE=1 SV=2 | 10.8420102 | 3.07486631 | 0.664665787 | 0.00257429 | 0.0832881 |
| O35143 | ATPase inhibitor, mitochondrial OS=Mus musculus OX=10090 GN=ATP5IF1 PE=1 SV=2 | 24.59631937 | 26.41509434 | 0.674873027 | 0.00387232 | 0.0989088 |
| P63168 | Dynein light chain 1, cytoplasmic OS=Mus musculus OX=10090 GN=Dynll1 PE=1 SV=1 | 133.8068602 | 57.30337079 | 0.720856501 | 0.0135923 | 0.20225 |
| P04919 | Band 3 anion transport protein OS=Mus musculus OX=10090 GN=Slc4a1 PE=1 SV=1 | 15.4338914 | 6.566200215 | 0.72440379 | 0.0301757 | 0.315196 |
| Q80XA6 | RalBP1-associated Eps domain-containing protein 2 OS=Mus musculus OX=10090 GN=Reps2 PE=1 SV=1 | 46.57157657 | 16.89059501 | 0.728455627 | 0.0349279 | 0.344313 |
| P02088 | Hemoglobin subunit beta-1 OS=Mus musculus OX=10090 GN=Hbb-b1 PE=1 SV=2 | 292.303841 | 75.51020408 | 0.730840759 | 7.28E-05 | 0.0163983 |
| Q60870 | Receptor expression-enhancing protein 5 OS=Mus musculus OX=10090 GN=Reep5 PE=1 SV=1 | 23.09445399 | 10.27027027 | 0.733952193 | 0.0216658 | 0.263879 |
| O09114 | Prostaglandin-H2 D-isomerase OS=Mus musculus OX=10090 GN=Ptgds PE=1 SV=1 | 24.94439466 | 13.75661376 | 0.737535808 | 0.00506013 | 0.117802 |
| Q9WVI9 | C-Jun-amino-terminal kinase-interacting protein 1 OS=Mus musculus OX=10090 GN=Mapk8ip1 PE=1 SV=2 | 5.362525281 | 2.404526167 | 0.738571245 | 0.0337857 | 0.337269 |
| P32848 | Parvalbumin alpha OS=Mus musculus OX=10090 GN=Pvalb PE=1 SV=3 | 49.99371517 | 49.09090909 | 0.739051087 | 0.0196587 | 0.247559 |
| Q91WM1 | Spermatid perinuclear RNA-binding protein OS=Mus musculus OX=10090 GN=Strbp PE=1 SV=1 | 27.27811879 | 11.01190476 | 0.742225257 | 0.0434772 | 0.381499 |
| P02463 | Collagen alpha-1(IV) chain OS=Mus musculus OX=10090 GN=Col4a1 PE=1 SV=4 | 25.93366777 | 3.235470342 | 0.75699412 | 0.000613816 | 0.0445122 |
| Q3U2I3 | FTS and Hook-interacting protein OS=Mus musculus OX=10090 GN=Fam160a2 PE=1 SV=2 | 27.86358828 | 11.69230769 | 0.762859353 | 0.00878013 | 0.158268 |
| Q3TWI9 | CSC1-like protein 2 OS=Mus musculus OX=10090 GN=Tmem63b PE=1 SV=1 | 7.704398615 | 2.403846154 | 0.763883217 | 0.043588 | 0.380355 |
| P06837 | Neuromodulin OS=Mus musculus OX=10090 GN=Gap43 PE=1 SV=1 | 224.0421129 | 57.26872247 | 0.775263152 | 0.000437224 | 0.0388513 |
| Q00897 | Alpha-1-antitrypsin 1-4 OS=Mus musculus OX=10090 GN=Serpina1d PE=1 SV=1 | 48.83049246 | 25.18159806 | 0.778534026 | 0.0137662 | 0.20245 |
| P63040 | Complexin-1 OS=Mus musculus OX=10090 GN=Cplx1 PE=1 SV=1 | 163.4232152 | 51.49253731 | 0.780624691 | 0.00197854 | 0.0734273 |
| P13634 | Carbonic anhydrase 1 OS=Mus musculus OX=10090 GN=Ca1 PE=1 SV=4 | 16.85196265 | 12.64367816 | 0.782924991 | 0.000586514 | 0.0440514 |
| O08677 | Kininogen-1 OS=Mus musculus OX=10090 GN=Kng1 PE=1 SV=1 | 26.23954647 | 10.89258699 | 0.783454828 | 0.0185504 | 0.239334 |
| Q8BZZ3 | NEDD4-like E3 ubiquitin-protein ligase WWP1 OS=Mus musculus OX=10090 GN=Wwp1 PE=1 SV=2 | 9.871744294 | 5.446623094 | 0.793996746 | 0.0468826 | 0.395431 |
| Q9Z2H2 | Regulator of G-protein signaling 6 OS=Mus musculus OX=10090 GN=Rgs6 PE=1 SV=2 | 59.1204258 | 23.94067797 | 0.794907558 | 0.0284775 | 0.305034 |
| P54227 | Stathmin OS=Mus musculus OX=10090 GN=Stmn1 PE=1 SV=2 | 66.5587213 | 44.96644295 | 0.796717959 | 0.00196451 | 0.0733377 |
| Q9JKC6 | Cell cycle exit and neuronal differentiation protein 1 OS=Mus musculus OX=10090 GN=Cend1 PE=1 SV=1 | 91.20116514 | 51.67785235 | 0.797332842 | 0.0271809 | 0.298233 |
| Q80ZX8 | Sperm-associated antigen 1 OS=Mus musculus OX=10090 GN=Spag1 PE=1 SV=1 | 4.421017157 | 1.886792453 | 0.79827404 | 0.0259365 | 0.289617 |
| Q91X72 | Hemopexin OS=Mus musculus OX=10090 GN=Hpx PE=1 SV=2 | 32.97280066 | 14.56521739 | 0.799825953 | 0.0049094 | 0.114716 |
| P17095 | High mobility group protein HMG-I/HMG-Y OS=Mus musculus OX=10090 GN=Hmga1 PE=1 SV=4 | 8.821130631 | 30.8411215 | 0.801271462 | 0.00352051 | 0.0953257 |
| P50428 | Arylsulfatase A OS=Mus musculus OX=10090 GN=Arsa PE=1 SV=2 | 11.40734089 | 7.90513834 | 0.802135017 | 0.00246373 | 0.0813804 |
| Q80TB8 | Synaptic vesicle membrane protein VAT-1 homolog-like OS=Mus musculus OX=10090 GN=Vat1l PE=1 SV=2 | 87.16048845 | 26.85851319 | 0.809709888 | 0.0140105 | 0.204612 |
| Q61838 | Pregnancy zone protein OS=Mus musculus OX=10090 GN=Pzp PE=1 SV=3 | 48.91485672 | 9.163879599 | 0.810273577 | 0.0106051 | 0.177945 |
| P07724 | Serum albumin OS=Mus musculus OX=10090 GN=Alb PE=1 SV=3 | 604.5728289 | 74.17763158 | 0.811629383 | 0.00780423 | 0.150113 |
| Q6PHZ2 | Calcium/calmodulin-dependent protein kinase type II subunit delta OS=Mus musculus OX=10090 GN=Camk2d PE=1 SV=1 | 249.7979057 | 49.09819639 | 0.816508042 | 0.0361881 | 0.350708 |
| P35802 | Neuronal membrane glycoprotein M6-a OS=Mus musculus OX=10090 GN=Gpm6a PE=1 SV=1 | 160.6664549 | 25.53956835 | 0.821838601 | 0.0160988 | 0.218424 |
| Q8BH86 | D-glutamate cyclase, mitochondrial OS=Mus musculus OX=10090 GN=Dglucy PE=1 SV=1 | 15.13364978 | 10.21069692 | 0.823381535 | 0.00117204 | 0.055182 |
| Q9JMK2 | Casein kinase I isoform epsilon OS=Mus musculus OX=10090 GN=Csnk1e PE=1 SV=2 | 25.74761175 | 17.78846154 | 0.823866222 | 0.0253513 | 0.286633 |
| P58802 | TBC1 domain family member 10A OS=Mus musculus OX=10090 GN=Tbc1d10a PE=1 SV=1 | 25.90210414 | 10.8 | 0.829873877 | 0.020607 | 0.253924 |
| Q8VDP6 | CDP-diacylglycerol--inositol 3-phosphatidyltransferase OS=Mus musculus OX=10090 GN=Cdipt PE=1 SV=1 | 18.38508338 | 15.96244131 | 1.200236742 | 0.0437584 | 0.379741 |
| Q68ED7 | CREB-regulated transcription coactivator 1 OS=Mus musculus OX=10090 GN=Crtc1 PE=1 SV=1 | 47.45443453 | 16.50793651 | 1.205369111 | 3.67E-05 | 0.0154561 |
| Q8VHW2 | Voltage-dependent calcium channel gamma-8 subunit OS=Mus musculus OX=10090 GN=Cacng8 PE=1 SV=1 | 84.79429237 | 34.75177305 | 1.211051318 | 0.0421044 | 0.375724 |
| Q8BI08 | Protein MAL2 OS=Mus musculus OX=10090 GN=Mal2 PE=1 SV=1 | 10.45739116 | 6.285714286 | 1.212522905 | 0.00267452 | 0.0827136 |
| P61971 | Nuclear transport factor 2 OS=Mus musculus OX=10090 GN=Nutf2 PE=1 SV=1 | 23.89890031 | 38.58267717 | 1.213600849 | 3.42E-05 | 0.0153976 |
| Q9Z140 | Copine-6 OS=Mus musculus OX=10090 GN=Cpne6 PE=1 SV=1 | 211.323338 | 43.62657092 | 1.214167953 | 0.00563815 | 0.124374 |
| Q9JJV5 | Voltage-dependent calcium channel gamma-3 subunit OS=Mus musculus OX=10090 GN=Cacng3 PE=1 SV=2 | 2.9076303 | 2.857142857 | 1.216252694 | 0.0162107 | 0.219471 |
| Q6WVG3 | BTB/POZ domain-containing protein KCTD12 OS=Mus musculus OX=10090 GN=Kctd12 PE=1 SV=1 | 128.4210048 | 51.68195719 | 1.225903145 | 5.37E-05 | 0.0153881 |
| Q3UX10 | Tubulin alpha chain-like 3 OS=Mus musculus OX=10090 GN=Tubal3 PE=2 SV=2 | 56.94562492 | 10.0896861 | 1.2273613 | 0.00647235 | 0.134323 |
| Q9CWU6 | Ubiquinol-cytochrome-c reductase complex assembly factor 1 OS=Mus musculus OX=10090 GN=Uqcc1 PE=1 SV=1 | 8.184732259 | 9.152542373 | 1.231396182 | 0.0427388 | 0.378707 |
| P62245 | 40S ribosomal protein S15a OS=Mus musculus OX=10090 GN=Rps15a PE=1 SV=2 | 24.27297064 | 30.76923077 | 1.236711511 | 0.00154456 | 0.0636905 |
| Q91VK4 | Integral membrane protein 2C OS=Mus musculus OX=10090 GN=Itm2c PE=1 SV=2 | 46.17330267 | 46.8401487 | 1.242587115 | 0.00268731 | 0.0823021 |
| P56379 | ATP synthase subunit ATP5MPL, mitochondrial OS=Mus musculus OX=10090 GN=Atp5mpl PE=1 SV=1 | 5.411826034 | 13.79310345 | 1.250434943 | 0.014063 | 0.203029 |
| Q9CQ89 | Protein CutA OS=Mus musculus OX=10090 GN=Cuta PE=1 SV=3 | 13.1610701 | 7.90960452 | 1.250631708 | 0.0278488 | 0.300339 |
| Q9JJY3 | Sphingomyelin phosphodiesterase 3 OS=Mus musculus OX=10090 GN=Smpd3 PE=1 SV=1 | 57.9449833 | 19.08396947 | 1.251140665 | 0.0214016 | 0.261166 |
| Q9Z239 | Phospholemman OS=Mus musculus OX=10090 GN=Fxyd1 PE=1 SV=1 | 12.61940243 | 13.04347826 | 1.258970955 | 0.0241176 | 0.279189 |
| P17665 | Cytochrome c oxidase subunit 7C, mitochondrial OS=Mus musculus OX=10090 GN=Cox7c PE=1 SV=1 | 16.26904069 | 28.57142857 | 1.267587203 | 0.019598 | 0.247287 |
| Q9R257 | Heme-binding protein 1 OS=Mus musculus OX=10090 GN=Hebp1 PE=1 SV=2 | 57.42776649 | 34.73684211 | 1.270013647 | 0.000292686 | 0.0307759 |
| P43024 | Cytochrome c oxidase subunit 6A1, mitochondrial OS=Mus musculus OX=10090 GN=Cox6a1 PE=1 SV=2 | 22.85224265 | 59.45945946 | 1.277467865 | 0.00980351 | 0.171331 |
| Q8CGA4 | Maturin OS=Mus musculus OX=10090 GN=Mturn PE=1 SV=1 | 17.89876478 | 22.13740458 | 1.281033319 | 0.0228156 | 0.275226 |
| P56382 | ATP synthase subunit epsilon, mitochondrial OS=Mus musculus OX=10090 GN=Atp5f1e PE=1 SV=2 | 10.44743486 | 44.23076923 | 1.282703743 | 0.000896784 | 0.0483574 |
| Q922F4 | Tubulin beta-6 chain OS=Mus musculus OX=10090 GN=Tubb6 PE=1 SV=1 | 316.7077414 | 44.51901566 | 1.282952716 | 0.000297285 | 0.030747 |
| Q8VCY8 | Phospholipid phosphatase-related protein type 2 OS=Mus musculus OX=10090 GN=Plppr2 PE=1 SV=1 | 28.9115601 | 18.36734694 | 1.285364916 | 0.00914748 | 0.163953 |
| P68373 | Tubulin alpha-1C chain OS=Mus musculus OX=10090 GN=Tuba1c PE=1 SV=1 | 667.8157434 | 69.48775056 | 1.288889873 | 0.00648369 | 0.134117 |
| P97478 | 5-demethoxyubiquinone hydroxylase, mitochondrial OS=Mus musculus OX=10090 GN=Coq7 PE=1 SV=3 | 3.013676223 | 4.147465438 | 1.299415663 | 0.00229413 | 0.0786613 |
| Q9D7X1 | BTB/POZ domain-containing protein KCTD4 OS=Mus musculus OX=10090 GN=Kctd4 PE=1 SV=1 | 52.14052639 | 33.59073359 | 1.302878002 | 0.00605821 | 0.129126 |
| P43274 | Histone H1.4 OS=Mus musculus OX=10090 GN=Hist1h1e PE=1 SV=2 | 47.61523593 | 21.91780822 | 1.324748047 | 0.0344962 | 0.342196 |
| P63087 | Serine/threonine-protein phosphatase PP1-gamma catalytic subunit OS=Mus musculus OX=10090 GN=Ppp1cc PE=1 SV=1 | 144.4957084 | 41.17647059 | 1.338439567 | 0.00176777 | 0.0697055 |
| Q9D5S7 | Leucine-rich repeat and guanylate kinase domain-containing protein OS=Mus musculus OX=10090 GN=Lrguk PE=1 SV=1 | 1.562090964 | 0.731707317 | 1.367178865 | 0.000658781 | 0.0446909 |
| P09240 | Cholecystokinin OS=Mus musculus OX=10090 GN=Cck PE=1 SV=3 | 5.584493132 | 14.7826087 | 1.388553727 | 0.000779171 | 0.045099 |
| P62077 | Mitochondrial import inner membrane translocase subunit Tim8 B OS=Mus musculus OX=10090 GN=Timm8b PE=1 SV=1 | 12.4629071 | 26.5060241 | 1.429957233 | 0.00283179 | 0.085482 |
| P63154 | Crooked neck-like protein 1 OS=Mus musculus OX=10090 GN=Crnkl1 PE=1 SV=1 | 7.550889515 | 3.333333333 | 1.470464546 | 3.84E-07 | 0.00242558 |
