# Supplementary material for: Effects of long-term low dose saxitoxin exposure on nerve damage in mice
Source: Aging (Albany NY). 2021 Jul 1;13(13):17211–26. doi: 10.18632/aging.203199 (PMC8312470; doi:10.18632/aging.203199)
Supplement: Supplementary Table 2 [file aging-13-203199-s002.docx]

**Supplementary Table 2. 87 proteins differentially expressed in middle compared with CT.**

| **Accession** | **Description** | **Sum PEP Score** | **Coverage** | **Fold Change middle_ct** | **p-value middle_ct** | **q-value middle_ct** |
| --- | --- | --- | --- | --- | --- | --- |
| P11679 | Keratin, type II cytoskeletal 8 OS=Mus musculus OX=10090 GN=Krt8 PE=1 SV=4 | 84.93853715 | 37.95918367 | 0.556466985 | 0.0325665 | 0.347064 |
| P02088 | Hemoglobin subunit beta-1 OS=Mus musculus OX=10090 GN=Hbb-b1 PE=1 SV=2 | 292.303841 | 75.51020408 | 0.647784117 | 2.48E-06 | 0.0156674 |
| P46097 | Synaptotagmin-2 OS=Mus musculus OX=10090 GN=Syt2 PE=1 SV=1 | 151.2727572 | 33.6492891 | 0.671116438 | 0.0202367 | 0.28184 |
| P07724 | Serum albumin OS=Mus musculus OX=10090 GN=Alb PE=1 SV=3 | 604.5728289 | 74.17763158 | 0.685671755 | 5.56E-05 | 0.0319092 |
| Q61838 | Pregnancy zone protein OS=Mus musculus OX=10090 GN=Pzp PE=1 SV=3 | 48.91485672 | 9.163879599 | 0.688599004 | 9.14E-05 | 0.0303427 |
| Q00623 | Apolipoprotein A-I OS=Mus musculus OX=10090 GN=Apoa1 PE=1 SV=2 | 87.95814894 | 48.10606061 | 0.691465895 | 0.000264402 | 0.0417029 |
| Q80TB8 | Synaptic vesicle membrane protein VAT-1 homolog-like OS=Mus musculus OX=10090 GN=Vat1l PE=1 SV=2 | 87.16048845 | 26.85851319 | 0.701516042 | 0.00138833 | 0.0867227 |
| Q8VDT9 | 39S ribosomal protein L50, mitochondrial OS=Mus musculus OX=10090 GN=Mrpl50 PE=1 SV=2 | 6.46005618 | 15.09433962 | 0.703686626 | 0.00190659 | 0.0985955 |
| O08677 | Kininogen-1 OS=Mus musculus OX=10090 GN=Kng1 PE=1 SV=1 | 26.23954647 | 10.89258699 | 0.714319792 | 0.00180284 | 0.0955808 |
| P04919 | Band 3 anion transport protein OS=Mus musculus OX=10090 GN=Slc4a1 PE=1 SV=1 | 15.4338914 | 6.566200215 | 0.715611759 | 0.0214186 | 0.28874 |
| P13634 | Carbonic anhydrase 1 OS=Mus musculus OX=10090 GN=Ca1 PE=1 SV=4 | 16.85196265 | 12.64367816 | 0.717578216 | 6.16E-05 | 0.0298756 |
| Q91X72 | Hemopexin OS=Mus musculus OX=10090 GN=Hpx PE=1 SV=2 | 32.97280066 | 14.56521739 | 0.728142135 | 0.000413933 | 0.0492737 |
| Q9Z0F7 | Gamma-synuclein OS=Mus musculus OX=10090 GN=Sncg PE=1 SV=1 | 48.55182035 | 61.78861789 | 0.733723806 | 0.0115542 | 0.211291 |
| P01942 | Hemoglobin subunit alpha OS=Mus musculus OX=10090 GN=Hba PE=1 SV=2 | 181.2265749 | 62.67605634 | 0.740284127 | 0.000448616 | 0.0505414 |
| Q9QXE0 | 2-hydroxyacyl-CoA lyase 1 OS=Mus musculus OX=10090 GN=Hacl1 PE=1 SV=2 | 9.034036012 | 4.647160069 | 0.749833403 | 0.00481795 | 0.153517 |
| P09470 | Angiotensin-converting enzyme OS=Mus musculus OX=10090 GN=Ace PE=1 SV=3 | 30.38965478 | 7.698170732 | 0.750606142 | 0.0318314 | 0.345059 |
| P32261 | Antithrombin-III OS=Mus musculus OX=10090 GN=Serpinc1 PE=1 SV=1 | 9.959869132 | 6.021505376 | 0.759305309 | 0.032316 | 0.347921 |
| Q08331 | Calretinin OS=Mus musculus OX=10090 GN=Calb2 PE=1 SV=3 | 136.264622 | 58.67158672 | 0.761964136 | 0.0354009 | 0.358498 |
| Q9CW46 | Ribonucleoprotein PTB-binding 1 OS=Mus musculus OX=10090 GN=Raver1 PE=1 SV=2 | 10.8420102 | 3.07486631 | 0.763098396 | 0.02967 | 0.334862 |
| Q00897 | Alpha-1-antitrypsin 1-4 OS=Mus musculus OX=10090 GN=Serpina1d PE=1 SV=1 | 48.83049246 | 25.18159806 | 0.763511081 | 0.0114032 | 0.210359 |
| P01872 | Immunoglobulin heavy constant mu OS=Mus musculus OX=10090 GN=Ighm PE=1 SV=2 | 33.07979589 | 13.43612335 | 0.765341746 | 0.00549968 | 0.166017 |
| P01027 | Complement C3 OS=Mus musculus OX=10090 GN=C3 PE=1 SV=3 | 89.48794082 | 12.56764883 | 0.767665118 | 2.70E-05 | 0.0284242 |
| P32848 | Parvalbumin alpha OS=Mus musculus OX=10090 GN=Pvalb PE=1 SV=3 | 49.99371517 | 49.09090909 | 0.768938969 | 0.0313707 | 0.343608 |
| P63168 | Dynein light chain 1, cytoplasmic OS=Mus musculus OX=10090 GN=Dynll1 PE=1 SV=1 | 133.8068602 | 57.30337079 | 0.772458322 | 0.0293948 | 0.333547 |
| P02463 | Collagen alpha-1(IV) chain OS=Mus musculus OX=10090 GN=Col4a1 PE=1 SV=4 | 25.93366777 | 3.235470342 | 0.773850079 | 5.89E-05 | 0.0309927 |
| Q3U2I3 | FTS and Hook-interacting protein OS=Mus musculus OX=10090 GN=Fam160a2 PE=1 SV=2 | 27.86358828 | 11.69230769 | 0.775535109 | 0.0115557 | 0.210707 |
| Q9CRB6 | Tubulin polymerization-promoting protein family member 3 OS=Mus musculus OX=10090 GN=Tppp3 PE=1 SV=1 | 60.06769363 | 44.31818182 | 0.781151347 | 0.0482021 | 0.409848 |
| P07356 | Annexin A2 OS=Mus musculus OX=10090 GN=Anxa2 PE=1 SV=2 | 91.68315359 | 35.39823009 | 0.785446001 | 0.00803337 | 0.191255 |
| Q8VCM7 | Fibrinogen gamma chain OS=Mus musculus OX=10090 GN=Fgg PE=1 SV=1 | 8.658400321 | 7.339449541 | 0.787706999 | 0.00831113 | 0.192775 |
| O09114 | Prostaglandin-H2 D-isomerase OS=Mus musculus OX=10090 GN=Ptgds PE=1 SV=1 | 24.94439466 | 13.75661376 | 0.78983653 | 0.0287895 | 0.332053 |
| Q921I1 | Serotransferrin OS=Mus musculus OX=10090 GN=Tf PE=1 SV=1 | 266.4427159 | 47.77618364 | 0.798785473 | 0.000537222 | 0.0546667 |
| P06837 | Neuromodulin OS=Mus musculus OX=10090 GN=Gap43 PE=1 SV=1 | 224.0421129 | 57.26872247 | 0.798928334 | 0.003993 | 0.13766 |
| Q61292 | Laminin subunit beta-2 OS=Mus musculus OX=10090 GN=Lamb2 PE=1 SV=2 | 182.8165346 | 23.40188994 | 0.808278848 | 1.56E-05 | 0.0197001 |
| Q9JKC6 | Cell cycle exit and neuronal differentiation protein 1 OS=Mus musculus OX=10090 GN=Cend1 PE=1 SV=1 | 91.20116514 | 51.67785235 | 0.810020317 | 0.0122379 | 0.21749 |
| Q9R069 | Basal cell adhesion molecule OS=Mus musculus OX=10090 GN=Bcam PE=1 SV=1 | 58.56461516 | 24.59807074 | 0.811530375 | 0.000249753 | 0.0414656 |
| Q62413 | Ephrin type-A receptor 6 OS=Mus musculus OX=10090 GN=Epha6 PE=1 SV=2 | 27.39621032 | 6.763285024 | 0.812492837 | 0.00963808 | 0.198714 |
| Q8BTM8 | Filamin-A OS=Mus musculus OX=10090 GN=Flna PE=1 SV=5 | 195.2732982 | 20.9671326 | 0.817116109 | 0.00723251 | 0.183992 |
| Q9JHU9 | Inositol-3-phosphate synthase 1 OS=Mus musculus OX=10090 GN=Isyna1 PE=1 SV=1 | 12.72589513 | 9.156193896 | 0.818101641 | 0.00627017 | 0.175038 |
| Q8K0E8 | Fibrinogen beta chain OS=Mus musculus OX=10090 GN=Fgb PE=1 SV=1 | 31.418748 | 15.59251559 | 0.819690396 | 0.0299947 | 0.33732 |
| P50428 | Arylsulfatase A OS=Mus musculus OX=10090 GN=Arsa PE=1 SV=2 | 11.40734089 | 7.90513834 | 0.820131412 | 0.00206603 | 0.103449 |
| O35668 | Huntingtin-associated protein 1 OS=Mus musculus OX=10090 GN=Hap1 PE=1 SV=1 | 23.2525269 | 8.439490446 | 0.820497589 | 0.00871474 | 0.190907 |
| P08122 | Collagen alpha-2(IV) chain OS=Mus musculus OX=10090 GN=Col4a2 PE=1 SV=4 | 38.69043181 | 3.925014646 | 0.820994237 | 0.0157215 | 0.246733 |
| Q9Z2H2 | Regulator of G-protein signaling 6 OS=Mus musculus OX=10090 GN=Rgs6 PE=1 SV=2 | 59.1204258 | 23.94067797 | 0.828211419 | 0.0463146 | 0.399179 |
| E9PV24 | Fibrinogen alpha chain OS=Mus musculus OX=10090 GN=Fga PE=1 SV=1 | 42.82637098 | 11.66032953 | 0.829089067 | 0.00895663 | 0.192858 |
| Q02357 | Ankyrin-1 OS=Mus musculus OX=10090 GN=Ank1 PE=1 SV=2 | 110.1468713 | 15.30612245 | 0.830022298 | 0.0108949 | 0.204572 |
| Q8BH86 | D-glutamate cyclase, mitochondrial OS=Mus musculus OX=10090 GN=Dglucy PE=1 SV=1 | 15.13364978 | 10.21069692 | 0.831194492 | 0.000591706 | 0.0548981 |
| Q3URS9 | Coiled-coil domain-containing protein 51 OS=Mus musculus OX=10090 GN=Ccdc51 PE=1 SV=1 | 29.65548017 | 14.77832512 | 0.832053961 | 0.00766762 | 0.189706 |
| P48036 | Annexin A5 OS=Mus musculus OX=10090 GN=Anxa5 PE=1 SV=1 | 147.7713107 | 65.20376176 | 0.832495281 | 0.00312445 | 0.12476 |
| Q6PIE5 | Sodium/potassium-transporting ATPase subunit alpha-2 OS=Mus musculus OX=10090 GN=Atp1a2 PE=1 SV=1 | 1055.895584 | 56.56862745 | 0.832711123 | 0.00739222 | 0.187299 |
| P58802 | TBC1 domain family member 10A OS=Mus musculus OX=10090 GN=Tbc1d10a PE=1 SV=1 | 25.90210414 | 10.8 | 0.832897577 | 0.0263273 | 0.314581 |
| Q3UHH2 | Solute carrier family 22 member 23 OS=Mus musculus OX=10090 GN=Slc22a23 PE=2 SV=1 | 11.21122539 | 3.193033382 | 0.835644369 | 0.0174561 | 0.26347 |
| Q8K406 | Leucine-rich repeat LGI family member 3 OS=Mus musculus OX=10090 GN=Lgi3 PE=1 SV=1 | 53.64169307 | 21.53284672 | 0.836345525 | 0.0393853 | 0.369764 |
| Q80YN3 | Breast carcinoma-amplified sequence 1 homolog OS=Mus musculus OX=10090 GN=Bcas1 PE=1 SV=3 | 160.5222999 | 22.11690363 | 0.836789701 | 0.00896656 | 0.191763 |
| P02468 | Laminin subunit gamma-1 OS=Mus musculus OX=10090 GN=Lamc1 PE=1 SV=2 | 132.7177326 | 15.86807716 | 0.836905712 | 0.000124513 | 0.0290947 |
| Q9DAS9 | Guanine nucleotide-binding protein G(I)/G(S)/G(O) subunit gamma-12 OS=Mus musculus OX=10090 GN=Gng12 PE=1 SV=3 | 86.28938812 | 54.16666667 | 0.836993892 | 0.000136211 | 0.0296329 |
| P17095 | High mobility group protein HMG-I/HMG-Y OS=Mus musculus OX=10090 GN=Hmga1 PE=1 SV=4 | 8.821130631 | 30.8411215 | 0.837293308 | 0.013418 | 0.226348 |
| P00920 | Carbonic anhydrase 2 OS=Mus musculus OX=10090 GN=Ca2 PE=1 SV=4 | 232.3597708 | 62.69230769 | 0.837700825 | 0.00453763 | 0.149104 |
| Q3TH73 | Protein tweety homolog 2 OS=Mus musculus OX=10090 GN=Ttyh2 PE=1 SV=1 | 7.738149758 | 4.69924812 | 0.838046383 | 0.00239203 | 0.108571 |
| Q8BI08 | Protein MAL2 OS=Mus musculus OX=10090 GN=Mal2 PE=1 SV=1 | 10.45739116 | 6.285714286 | 1.204956446 | 0.00306639 | 0.123222 |
| Q8CBW3 | Abl interactor 1 OS=Mus musculus OX=10090 GN=Abi1 PE=1 SV=3 | 96.6431171 | 35.34303534 | 1.211277987 | 0.00261025 | 0.114361 |
| P45377 | Aldose reductase-related protein 2 OS=Mus musculus OX=10090 GN=Akr1b8 PE=1 SV=2 | 19.76100751 | 11.07594937 | 1.211908686 | 0.00861753 | 0.19348 |
| Q91VK4 | Integral membrane protein 2C OS=Mus musculus OX=10090 GN=Itm2c PE=1 SV=2 | 46.17330267 | 46.8401487 | 1.212241384 | 0.00579323 | 0.171594 |
| Q80TN4 | DnaJ homolog subfamily C member 16 OS=Mus musculus OX=10090 GN=Dnajc16 PE=1 SV=2 | 11.8755867 | 7.772020725 | 1.214445712 | 0.0413881 | 0.376793 |
| O35493 | Dual specificity protein kinase CLK4 OS=Mus musculus OX=10090 GN=Clk4 PE=1 SV=1 | 3.451199947 | 3.118503119 | 1.218848658 | 0.0141061 | 0.234199 |
| P97478 | 5-demethoxyubiquinone hydroxylase, mitochondrial OS=Mus musculus OX=10090 GN=Coq7 PE=1 SV=3 | 3.013676223 | 4.147465438 | 1.220050614 | 0.00207088 | 0.102072 |
| Q8R5H6 | Wiskott-Aldrich syndrome protein family member 1 OS=Mus musculus OX=10090 GN=Wasf1 PE=1 SV=2 | 155.3888058 | 32.73703041 | 1.220119962 | 0.0377428 | 0.364655 |
| P84075 | Neuron-specific calcium-binding protein hippocalcin OS=Mus musculus OX=10090 GN=Hpca PE=1 SV=2 | 188.780869 | 68.9119171 | 1.220547126 | 0.0261208 | 0.313897 |
| Q9JJY3 | Sphingomyelin phosphodiesterase 3 OS=Mus musculus OX=10090 GN=Smpd3 PE=1 SV=1 | 57.9449833 | 19.08396947 | 1.222108183 | 0.0316178 | 0.34452 |
| P14106 | Complement C1q subcomponent subunit B OS=Mus musculus OX=10090 GN=C1qb PE=1 SV=2 | 27.99155058 | 13.83399209 | 1.228700247 | 0.000348028 | 0.0448104 |
| Q9Z140 | Copine-6 OS=Mus musculus OX=10090 GN=Cpne6 PE=1 SV=1 | 211.323338 | 43.62657092 | 1.230302431 | 0.00799468 | 0.191781 |
| Q8VCY8 | Phospholipid phosphatase-related protein type 2 OS=Mus musculus OX=10090 GN=Plppr2 PE=1 SV=1 | 28.9115601 | 18.36734694 | 1.23167532 | 0.0182081 | 0.267774 |
| P62245 | 40S ribosomal protein S15a OS=Mus musculus OX=10090 GN=Rps15a PE=1 SV=2 | 24.27297064 | 30.76923077 | 1.237696852 | 0.00164062 | 0.0915988 |
| Q8VHW2 | Voltage-dependent calcium channel gamma-8 subunit OS=Mus musculus OX=10090 GN=Cacng8 PE=1 SV=1 | 84.79429237 | 34.75177305 | 1.239333102 | 0.0382062 | 0.366327 |
| Q9D5S7 | Leucine-rich repeat and guanylate kinase domain-containing protein OS=Mus musculus OX=10090 GN=Lrguk PE=1 SV=1 | 1.562090964 | 0.731707317 | 1.241512683 | 0.00147098 | 0.0883848 |
| P56379 | ATP synthase subunit ATP5MPL, mitochondrial OS=Mus musculus OX=10090 GN=Atp5mpl PE=1 SV=1 | 5.411826034 | 13.79310345 | 1.249288778 | 0.00799539 | 0.191072 |
| Q9R257 | Heme-binding protein 1 OS=Mus musculus OX=10090 GN=Hebp1 PE=1 SV=2 | 57.42776649 | 34.73684211 | 1.25375895 | 0.0019105 | 0.0979948 |
| P63087 | Serine/threonine-protein phosphatase PP1-gamma catalytic subunit OS=Mus musculus OX=10090 GN=Ppp1cc PE=1 SV=1 | 144.4957084 | 41.17647059 | 1.273686304 | 8.59E-05 | 0.0361103 |
| P30276 | G2/mitotic-specific cyclin-B2 OS=Mus musculus OX=10090 GN=Ccnb2 PE=1 SV=2 | 2.387746094 | 3.266331658 | 1.274188746 | 0.0230302 | 0.295922 |
| Q9D7X1 | BTB/POZ domain-containing protein KCTD4 OS=Mus musculus OX=10090 GN=Kctd4 PE=1 SV=1 | 52.14052639 | 33.59073359 | 1.295725397 | 0.0175879 | 0.264826 |
| P62077 | Mitochondrial import inner membrane translocase subunit Tim8 B OS=Mus musculus OX=10090 GN=Timm8b PE=1 SV=1 | 12.4629071 | 26.5060241 | 1.304598613 | 0.00257892 | 0.11458 |
| P09240 | Cholecystokinin OS=Mus musculus OX=10090 GN=Cck PE=1 SV=3 | 5.584493132 | 14.7826087 | 1.310674097 | 0.00289575 | 0.119407 |
| P63154 | Crooked neck-like protein 1 OS=Mus musculus OX=10090 GN=Crnkl1 PE=1 SV=1 | 7.550889515 | 3.333333333 | 1.318233556 | 7.12E-06 | 0.0149658 |
| S4R2P9 | Sodium/calcium exchanger 3 OS=Mus musculus OX=10090 GN=Slc8a3 PE=1 SV=1 | 16.6599283 | 3.017241379 | 1.327709019 | 0.0421492 | 0.381519 |
| Q8K3F6 | Potassium voltage-gated channel subfamily KQT member 3 OS=Mus musculus OX=10090 GN=Kcnq3 PE=1 SV=2 | 6.961072921 | 4.12371134 | 1.333911544 | 0.0329108 | 0.348379 |
| Q922F4 | Tubulin beta-6 chain OS=Mus musculus OX=10090 GN=Tubb6 PE=1 SV=1 | 316.7077414 | 44.51901566 | 1.352862297 | 6.63E-05 | 0.0298796 |
| Q6QI06 | Rapamycin-insensitive companion of mTOR OS=Mus musculus OX=10090 GN=Rictor PE=1 SV=2 | 3.897038978 | 0.819672131 | 1.373230685 | 0.0337237 | 0.352257 |
| P63084 | Protein S100-A5 OS=Mus musculus OX=10090 GN=S100a5 PE=3 SV=1 | 14.22364728 | 32.25806452 | 1.711784394 | 0.0403973 | 0.374253 |
