# Supplementary material for: Effects of long-term low dose saxitoxin exposure on nerve damage in mice
Source: Aging (Albany NY). 2021 Jul 1;13(13):17211–26. doi: 10.18632/aging.203199 (PMC8312470; doi:10.18632/aging.203199)
Supplement: Supplementary Table 3 [file aging-13-203199-s003.docx]

**Supplementary Table 3. 87 proteins differentially expressed in high compared with CT.**

| **Accession** | **Description** | **Sum PEP Score** | **Coverage** | **Fold Change high_ct** | **p-value high_ct** | **q-value high_ct** |
| --- | --- | --- | --- | --- | --- | --- |
| Q99J09 | Methylosome protein 50 OS=Mus musculus OX=10090 GN=Wdr77 PE=1 SV=1 | 35.77178456 | 31.57894737 | 1.201048991 | 0.0139078 | 0.23461 |
| Q6ZWV7 | 60S ribosomal protein L35 OS=Mus musculus OX=10090 GN=Rpl35 PE=1 SV=1 | 17.86184655 | 25.20325203 | 1.203604162 | 0.0112123 | 0.213068 |
| P46096 | Synaptotagmin-1 OS=Mus musculus OX=10090 GN=Syt1 PE=1 SV=1 | 388.7970346 | 63.18289786 | 1.204949764 | 0.0116408 | 0.214742 |
| Q4JIM5 | Tyrosine-protein kinase ABL2 OS=Mus musculus OX=10090 GN=Abl2 PE=1 SV=1 | 42.06243309 | 10.65989848 | 1.208863206 | 0.00788863 | 0.185016 |
| P63154 | Crooked neck-like protein 1 OS=Mus musculus OX=10090 GN=Crnkl1 PE=1 SV=1 | 7.550889515 | 3.333333333 | 1.209605832 | 0.0131588 | 0.229334 |
| P35283 | Ras-related protein Rab-12 OS=Mus musculus OX=10090 GN=Rab12 PE=1 SV=3 | 53.63209514 | 44.44444444 | 1.210722303 | 0.000287665 | 0.0625819 |
| P17665 | Cytochrome c oxidase subunit 7C, mitochondrial OS=Mus musculus OX=10090 GN=Cox7c PE=1 SV=1 | 16.26904069 | 28.57142857 | 1.217605685 | 0.0178226 | 0.263331 |
| Q8VCY8 | Phospholipid phosphatase-related protein type 2 OS=Mus musculus OX=10090 GN=Plppr2 PE=1 SV=1 | 28.9115601 | 18.36734694 | 1.220808574 | 0.0234841 | 0.304858 |
| P97478 | 5-demethoxyubiquinone hydroxylase, mitochondrial OS=Mus musculus OX=10090 GN=Coq7 PE=1 SV=3 | 3.013676223 | 4.147465438 | 1.221376507 | 0.00429002 | 0.140237 |
| P68373 | Tubulin alpha-1C chain OS=Mus musculus OX=10090 GN=Tuba1c PE=1 SV=1 | 667.8157434 | 69.48775056 | 1.222112418 | 0.00794414 | 0.183588 |
| P63087 | Serine/threonine-protein phosphatase PP1-gamma catalytic subunit OS=Mus musculus OX=10090 GN=Ppp1cc PE=1 SV=1 | 144.4957084 | 41.17647059 | 1.223884996 | 0.00234282 | 0.118247 |
| Q8BI08 | Protein MAL2 OS=Mus musculus OX=10090 GN=Mal2 PE=1 SV=1 | 10.45739116 | 6.285714286 | 1.224998515 | 0.0028696 | 0.125724 |
| Q922H4 | Mannose-1-phosphate guanyltransferase alpha OS=Mus musculus OX=10090 GN=Gmppa PE=1 SV=1 | 26.41272648 | 17.85714286 | 1.226455594 | 0.00472881 | 0.145532 |
| Q9JJY3 | Sphingomyelin phosphodiesterase 3 OS=Mus musculus OX=10090 GN=Smpd3 PE=1 SV=1 | 57.9449833 | 19.08396947 | 1.227198819 | 0.0271741 | 0.329696 |
| Q9JJV5 | Voltage-dependent calcium channel gamma-3 subunit OS=Mus musculus OX=10090 GN=Cacng3 PE=1 SV=2 | 2.9076303 | 2.857142857 | 1.227243904 | 0.0214582 | 0.29303 |
| Q9Z1T2 | Thrombospondin-4 OS=Mus musculus OX=10090 GN=Thbs4 PE=1 SV=1 | 13.64876807 | 6.645898235 | 1.228730056 | 0.0416342 | 0.397985 |
| Q8BP67 | 60S ribosomal protein L24 OS=Mus musculus OX=10090 GN=Rpl24 PE=1 SV=2 | 26.64045622 | 24.84076433 | 1.235842592 | 0.00115855 | 0.0891373 |
| P19253 | 60S ribosomal protein L13a OS=Mus musculus OX=10090 GN=Rpl13a PE=1 SV=4 | 29.381922 | 28.07881773 | 1.251442496 | 5.70E-05 | 0.0359657 |
| Q9CR57 | 60S ribosomal protein L14 OS=Mus musculus OX=10090 GN=Rpl14 PE=1 SV=3 | 23.57399519 | 15.66820276 | 1.254141386 | 0.00533389 | 0.154365 |
| P14148 | 60S ribosomal protein L7 OS=Mus musculus OX=10090 GN=Rpl7 PE=1 SV=2 | 76.80122562 | 22.22222222 | 1.254676122 | 0.000146553 | 0.0462302 |
| P45377 | Aldose reductase-related protein 2 OS=Mus musculus OX=10090 GN=Akr1b8 PE=1 SV=2 | 19.76100751 | 11.07594937 | 1.255454723 | 0.0205764 | 0.285311 |
| Q9CQR6 | Serine/threonine-protein phosphatase 6 catalytic subunit OS=Mus musculus OX=10090 GN=Ppp6c PE=1 SV=1 | 20.99280018 | 18.36065574 | 1.266723809 | 0.01557 | 0.241949 |
| P35922 | Synaptic functional regulator FMR1 OS=Mus musculus OX=10090 GN=Fmr1 PE=1 SV=1 | 43.17292556 | 17.752443 | 1.274627772 | 0.009369 | 0.198352 |
| Q9D5S7 | Leucine-rich repeat and guanylate kinase domain-containing protein OS=Mus musculus OX=10090 GN=Lrguk PE=1 SV=1 | 1.562090964 | 0.731707317 | 1.293457813 | 0.00241647 | 0.115496 |
| P62077 | Mitochondrial import inner membrane translocase subunit Tim8 B OS=Mus musculus OX=10090 GN=Timm8b PE=1 SV=1 | 12.4629071 | 26.5060241 | 1.296211375 | 0.00787858 | 0.186165 |
| P09240 | Cholecystokinin OS=Mus musculus OX=10090 GN=Cck PE=1 SV=3 | 5.584493132 | 14.7826087 | 1.298308291 | 0.00260489 | 0.118232 |
| P62245 | 40S ribosomal protein S15a OS=Mus musculus OX=10090 GN=Rps15a PE=1 SV=2 | 24.27297064 | 30.76923077 | 1.300156238 | 0.000493285 | 0.0662156 |
| Q922F4 | Tubulin beta-6 chain OS=Mus musculus OX=10090 GN=Tubb6 PE=1 SV=1 | 316.7077414 | 44.51901566 | 1.324992322 | 0.000310233 | 0.0652419 |
| P56379 | ATP synthase subunit ATP5MPL, mitochondrial OS=Mus musculus OX=10090 GN=Atp5mpl PE=1 SV=1 | 5.411826034 | 13.79310345 | 1.329897448 | 0.0041909 | 0.14064 |
| P43277 | Histone H1.3 OS=Mus musculus OX=10090 GN=Hist1h1d PE=1 SV=2 | 45.06173805 | 21.71945701 | 1.336419557 | 0.0182796 | 0.267578 |
| Q91VK4 | Integral membrane protein 2C OS=Mus musculus OX=10090 GN=Itm2c PE=1 SV=2 | 46.17330267 | 46.8401487 | 1.353120198 | 0.000802736 | 0.0803882 |
| Q8BYG9 | Ephrin type-A receptor 10 OS=Mus musculus OX=10090 GN=Epha10 PE=2 SV=2 | 14.74792405 | 2.879841112 | 1.651184119 | 0.0449121 | 0.414862 |
| P50446 | Keratin, type II cytoskeletal 6A OS=Mus musculus OX=10090 GN=Krt6a PE=1 SV=3 | 41.89014015 | 14.46654611 | 0.684341337 | 0.0441433 | 0.412593 |
| P02088 | Hemoglobin subunit beta-1 OS=Mus musculus OX=10090 GN=Hbb-b1 PE=1 SV=2 | 292.303841 | 75.51020408 | 0.691310623 | 4.45E-05 | 0.0312289 |
| P07724 | Serum albumin OS=Mus musculus OX=10090 GN=Alb PE=1 SV=3 | 604.5728289 | 74.17763158 | 0.698359597 | 0.000395763 | 0.0640223 |
| P46097 | Synaptotagmin-2 OS=Mus musculus OX=10090 GN=Syt2 PE=1 SV=1 | 151.2727572 | 33.6492891 | 0.699581964 | 0.0287839 | 0.341991 |
| P13634 | Carbonic anhydrase 1 OS=Mus musculus OX=10090 GN=Ca1 PE=1 SV=4 | 16.85196265 | 12.64367816 | 0.700091308 | 6.86E-05 | 0.0393436 |
| Q08331 | Calretinin OS=Mus musculus OX=10090 GN=Calb2 PE=1 SV=3 | 136.264622 | 58.67158672 | 0.714932033 | 0.0150502 | 0.239174 |
| Q9Z0F7 | Gamma-synuclein OS=Mus musculus OX=10090 GN=Sncg PE=1 SV=1 | 48.55182035 | 61.78861789 | 0.719833425 | 0.00877851 | 0.193649 |
| Q91X72 | Hemopexin OS=Mus musculus OX=10090 GN=Hpx PE=1 SV=2 | 32.97280066 | 14.56521739 | 0.723051343 | 0.000143372 | 0.0476069 |
| Q00623 | Apolipoprotein A-I OS=Mus musculus OX=10090 GN=Apoa1 PE=1 SV=2 | 87.95814894 | 48.10606061 | 0.724798563 | 0.0037027 | 0.135816 |
| P01872 | Immunoglobulin heavy constant mu OS=Mus musculus OX=10090 GN=Ighm PE=1 SV=2 | 33.07979589 | 13.43612335 | 0.729833359 | 0.0070675 | 0.17694 |
| P37804 | Transgelin OS=Mus musculus OX=10090 GN=Tagln PE=1 SV=3 | 53.84400246 | 48.25870647 | 0.732876495 | 0.0444467 | 0.41359 |
| P04919 | Band 3 anion transport protein OS=Mus musculus OX=10090 GN=Slc4a1 PE=1 SV=1 | 15.4338914 | 6.566200215 | 0.749919685 | 0.0397631 | 0.387736 |
| Q8VDT9 | 39S ribosomal protein L50, mitochondrial OS=Mus musculus OX=10090 GN=Mrpl50 PE=1 SV=2 | 6.46005618 | 15.09433962 | 0.755821781 | 0.0156569 | 0.240926 |
| Q9CRB6 | Tubulin polymerization-promoting protein family member 3 OS=Mus musculus OX=10090 GN=Tppp3 PE=1 SV=1 | 60.06769363 | 44.31818182 | 0.757343132 | 0.0241286 | 0.310035 |
| Q61838 | Pregnancy zone protein OS=Mus musculus OX=10090 GN=Pzp PE=1 SV=3 | 48.91485672 | 9.163879599 | 0.760709222 | 0.00296116 | 0.127959 |
| P32848 | Parvalbumin alpha OS=Mus musculus OX=10090 GN=Pvalb PE=1 SV=3 | 49.99371517 | 49.09090909 | 0.762314382 | 0.0315037 | 0.35429 |
| P01027 | Complement C3 OS=Mus musculus OX=10090 GN=C3 PE=1 SV=3 | 89.48794082 | 12.56764883 | 0.763082528 | 0.000349575 | 0.0668323 |
| O09114 | Prostaglandin-H2 D-isomerase OS=Mus musculus OX=10090 GN=Ptgds PE=1 SV=1 | 24.94439466 | 13.75661376 | 0.768870216 | 0.015181 | 0.238846 |
| Q8K0E8 | Fibrinogen beta chain OS=Mus musculus OX=10090 GN=Fgb PE=1 SV=1 | 31.418748 | 15.59251559 | 0.772452968 | 0.0106375 | 0.211709 |
| O08677 | Kininogen-1 OS=Mus musculus OX=10090 GN=Kng1 PE=1 SV=1 | 26.23954647 | 10.89258699 | 0.772773218 | 0.00343486 | 0.134599 |
| P63168 | Dynein light chain 1, cytoplasmic OS=Mus musculus OX=10090 GN=Dynll1 PE=1 SV=1 | 133.8068602 | 57.30337079 | 0.773128968 | 0.0216323 | 0.293501 |
| Q8VCM7 | Fibrinogen gamma chain OS=Mus musculus OX=10090 GN=Fgg PE=1 SV=1 | 8.658400321 | 7.339449541 | 0.773508473 | 0.00460453 | 0.143103 |
| Q9JL62 | Glycolipid transfer protein OS=Mus musculus OX=10090 GN=Gltp PE=1 SV=3 | 43.86364031 | 35.88516746 | 0.774341034 | 0.00426536 | 0.140891 |
| P08551 | Neurofilament light polypeptide OS=Mus musculus OX=10090 GN=Nefl PE=1 SV=5 | 354.6912977 | 60.40515654 | 0.782523509 | 0.0229223 | 0.302546 |
| Q9QXE0 | 2-hydroxyacyl-CoA lyase 1 OS=Mus musculus OX=10090 GN=Hacl1 PE=1 SV=2 | 9.034036012 | 4.647160069 | 0.790279012 | 0.0187285 | 0.271005 |
| Q3TH73 | Protein tweety homolog 2 OS=Mus musculus OX=10090 GN=Ttyh2 PE=1 SV=1 | 7.738149758 | 4.69924812 | 0.791183914 | 0.00079192 | 0.0832704 |
| Q80TB8 | Synaptic vesicle membrane protein VAT-1 homolog-like OS=Mus musculus OX=10090 GN=Vat1l PE=1 SV=2 | 87.16048845 | 26.85851319 | 0.792047029 | 0.0136563 | 0.234124 |
| P07356 | Annexin A2 OS=Mus musculus OX=10090 GN=Anxa2 PE=1 SV=2 | 91.68315359 | 35.39823009 | 0.79351093 | 0.00265008 | 0.119424 |
| Q80YN3 | Breast carcinoma-amplified sequence 1 homolog OS=Mus musculus OX=10090 GN=Bcas1 PE=1 SV=3 | 160.5222999 | 22.11690363 | 0.794341344 | 0.000285752 | 0.0643861 |
| Q921I1 | Serotransferrin OS=Mus musculus OX=10090 GN=Tf PE=1 SV=1 | 266.4427159 | 47.77618364 | 0.794697659 | 0.000673337 | 0.0801525 |
| Q9DAS9 | Guanine nucleotide-binding protein G(I)/G(S)/G(O) subunit gamma-12 OS=Mus musculus OX=10090 GN=Gng12 PE=1 SV=3 | 86.28938812 | 54.16666667 | 0.797379268 | 0.000467113 | 0.0640656 |
| Q922J6 | Tetraspanin-2 OS=Mus musculus OX=10090 GN=Tspan2 PE=1 SV=1 | 45.18401716 | 13.12217195 | 0.79787243 | 0.0205119 | 0.285672 |
| P20152 | Vimentin OS=Mus musculus OX=10090 GN=Vim PE=1 SV=3 | 307.9779934 | 66.9527897 | 0.799457918 | 0.00642581 | 0.168217 |
| P00920 | Carbonic anhydrase 2 OS=Mus musculus OX=10090 GN=Ca2 PE=1 SV=4 | 232.3597708 | 62.69230769 | 0.807641528 | 0.000691434 | 0.0752113 |
| Q9D0M5 | Dynein light chain 2, cytoplasmic OS=Mus musculus OX=10090 GN=Dynll2 PE=1 SV=1 | 149.7443237 | 57.30337079 | 0.80836065 | 0.00412025 | 0.139756 |
| Q9CR59 | Growth arrest and DNA damage-inducible proteins-interacting protein 1 OS=Mus musculus OX=10090 GN=Gadd45gip1 PE=1 SV=1 | 18.9674039 | 17.11711712 | 0.808714285 | 0.0422295 | 0.401849 |
| Q00897 | Alpha-1-antitrypsin 1-4 OS=Mus musculus OX=10090 GN=Serpina1d PE=1 SV=1 | 48.83049246 | 25.18159806 | 0.809469709 | 0.0313253 | 0.354178 |
| E9PV24 | Fibrinogen alpha chain OS=Mus musculus OX=10090 GN=Fga PE=1 SV=1 | 42.82637098 | 11.66032953 | 0.809493275 | 0.00792848 | 0.1839 |
| P01942 | Hemoglobin subunit alpha OS=Mus musculus OX=10090 GN=Hba PE=1 SV=2 | 181.2265749 | 62.67605634 | 0.812713069 | 0.0175705 | 0.26083 |
| P63040 | Complexin-1 OS=Mus musculus OX=10090 GN=Cplx1 PE=1 SV=1 | 163.4232152 | 51.49253731 | 0.814735118 | 0.0146727 | 0.240441 |
| Q9D154 | Leukocyte elastase inhibitor A OS=Mus musculus OX=10090 GN=Serpinb1a PE=1 SV=1 | 74.37401706 | 33.24538259 | 0.81553404 | 0.00214722 | 0.116783 |
| P48036 | Annexin A5 OS=Mus musculus OX=10090 GN=Anxa5 PE=1 SV=1 | 147.7713107 | 65.20376176 | 0.816641053 | 0.000934279 | 0.0879756 |
| P20917 | Myelin-associated glycoprotein OS=Mus musculus OX=10090 GN=Mag PE=1 SV=3 | 102.6696449 | 22.80701754 | 0.817991638 | 0.00345448 | 0.134533 |
| P46660 | Alpha-internexin OS=Mus musculus OX=10090 GN=Ina PE=1 SV=3 | 453.5619695 | 72.45508982 | 0.819235422 | 0.0101411 | 0.205065 |
| P50428 | Arylsulfatase A OS=Mus musculus OX=10090 GN=Arsa PE=1 SV=2 | 11.40734089 | 7.90513834 | 0.820288325 | 0.0111488 | 0.214445 |
| P60202 | Myelin proteolipid protein OS=Mus musculus OX=10090 GN=Plp1 PE=1 SV=2 | 119.30634 | 29.24187726 | 0.821496879 | 0.0320037 | 0.358634 |
| O08638 | Myosin-11 OS=Mus musculus OX=10090 GN=Myh11 PE=1 SV=1 | 395.8339642 | 28.60040568 | 0.823955884 | 0.0200285 | 0.281425 |
| P04370 | Myelin basic protein OS=Mus musculus OX=10090 GN=Mbp PE=1 SV=2 | 222.7086139 | 42.4 | 0.825357467 | 0.0190065 | 0.272527 |
| Q9CQ92 | Mitochondrial fission 1 protein OS=Mus musculus OX=10090 GN=Fis1 PE=1 SV=1 | 42.55132228 | 34.21052632 | 0.825523964 | 0.000112685 | 0.0394962 |
| P63254 | Cysteine-rich protein 1 OS=Mus musculus OX=10090 GN=Crip1 PE=1 SV=2 | 5.002751266 | 12.98701299 | 0.825679619 | 0.0199961 | 0.281597 |
| P24549 | Retinal dehydrogenase 1 OS=Mus musculus OX=10090 GN=Aldh1a1 PE=1 SV=5 | 62.2924775 | 30.33932136 | 0.826083774 | 0.0232544 | 0.304382 |
| Q8BH86 | D-glutamate cyclase, mitochondrial OS=Mus musculus OX=10090 GN=Dglucy PE=1 SV=1 | 15.13364978 | 10.21069692 | 0.829344264 | 8.02E-05 | 0.036157 |
| O54724 | Caveolae-associated protein 1 OS=Mus musculus OX=10090 GN=Cavin1 PE=1 SV=1 | 57.68830136 | 26.02040816 | 0.832379881 | 0.00568167 | 0.160025 |
| Q62426 | Cystatin-B OS=Mus musculus OX=10090 GN=Cstb PE=1 SV=1 | 29.34063195 | 54.08163265 | 0.83410216 | 0.00249169 | 0.116445 |
